# Experimental and clinical evidence of differential effects of magnesium sulfate on neuroprotection and angiogenesis in the fetal brain

**DOI:** 10.1002/prp2.315

**Published:** 2017-06-08

**Authors:** Matthieu Lecuyer, Marina Rubio, Clément Chollat, Maryline Lecointre, Sylvie Jégou, Philippe Leroux, Carine Cleren, Isabelle Leroux‐Nicollet, Loic Marpeau, Denis Vivien, Stéphane Marret, Bruno J. Gonzalez

**Affiliations:** ^1^ Normandie University UNIROUEN INSERM U1245 NeoVasc Team Rouen University Hospital IRIB F76000 Normandy Centre for Genomic and Personalized Medicine Rouen France; ^2^ INSERM U1237 unit “Serine proteases and Pathophysiology of the neurovascular Unit” Normandy University Caen France; ^3^ Department of Neonatal Paediatrics and Intensive Care Rouen Hospital Rouen France; ^4^ Department of Neonatal Intensive Care Port‐Royal University Hospital APHP Paris France; ^5^ Department of Obstetrics Rouen Hospital Rouen France

**Keywords:** Cerebral palsy, Hif, Neurovascular, side effects, translational medicine

## Abstract

Clinical studies showed beneficial effects of magnesium sulfate regarding the risk of cerebral palsy. However, regimen protocols fluctuate worldwide and risks of adverse effects impacting the vascular system have been reported for human neonates, keeping open the question of the optimal dosing. Using clinically relevant concentrations and doses of magnesium sulfate, experiments consisted of characterizing, respectively, ex vivo and in vivo, the effects of magnesium sulfate on the nervous and vascular systems of mouse neonates by targeting neuroprotection, angiogenesis, and hemodynamic factors and in measuring, in human fetuses, the impact of a 4‐g neuroprotective loading dose of magnesium sulfate on brain hemodynamic parameters. Preclinical experiments using cultured cortical slices from mouse neonates showed that the lowest and highest tested concentrations of magnesium sulfate were equally potent to prevent excitotoxic‐induced cell death, cell edema, cell burst, and intracellular calcium increase, whereas no side effects were found regarding apoptosis. In contrast, in vivo data revealed that magnesium sulfate exerted dose‐dependent vascular effects on the fetal brain. In particular, it induced brain hypoperfusion, stabilization of Hif‐1*α*, long‐term upregulation of VEGF‐R2 expression, impaired endothelial viability, and altered cortical angiogenesis. Clinically, in contrast to 6‐g loading doses used in some protocols, a 4‐g bolus of magnesium sulfate did not altered fetal brain hemodynamic parameters. In conclusion, these data provide the first mechanistic evidence of double‐sword and dose‐dependent actions of magnesium sulfate on nervous and vascular systems. They strongly support the clinical use of neuroprotection protocols validated for the lowest (4‐g) loading dose of magnesium sulfate.

**Abbreviations**
7‐AAD: 7‐aminoactinomycin D
aCS: artificial cerebrospinal fluid
CP: cerebral palsy
GD15: gestational day 15
LDH: lactate dehydrogenase
MCA: middle cerebral artery
MgSO_4_: magnesium sulfate
NADH: *β*‐nicotinamide adenine dinucleotide
NADPH: *β*‐nicotinamide adenine dinucleotide 2′‐phosphate reduced tetrasodium salt hydrate
NMRI: National Marine Research Institute
P2: postnatal day 2
PI: pulsatility index
PMSF: phenylmethanesulfonyl fluoride
RI: resistance index
ROI: region of interest
WG: week of gestation

## Introduction

Cerebral palsy (CP) is the most prevalent cause of motor dysfunction in neonates. It is defined as a group of permanent movement and posture disorders strongly associated with fetal or perinatal brain lesions (Volpe 2009). It affects both preterm and term neonates, even if the level of risk at birth is inversely related to gestational age (Nelson and Blair 2015). CP is associated with substantial healthcare and societal costs, as well as a significant reduction in health‐related quality of life for patients with moderate to severe disabilities (Koman et al. 2004).

Administration of magnesium sulfate (MgSO_4_) to pregnant women at risk for imminent preterm delivery has been shown to be neuroprotective for the fetus (Doyle et al. 2007) and several randomized controlled trials (Altman et al. 2002; Mittendorf et al. 2002; Crowther et al. 2003; Marret et al. 2007; Rouse et al. 2008), and meta‐analysis (Costantine and Weiner 2009; Zeng et al. 2016) revealed beneficial effects of MgSO_4_ regarding the risk of cerebral palsy. As a consequence, the antenatal use of MgSO_4_ is now recommended for neuroprotection by several colleges of obstetricians and gynecologists worldwide (Obstetric Care Consensus No. 3: Periviable Birth, 2015). Nevertheless, clinical studies have mentioned the possibility of adverse effects for preterm neonates with high serum magnesium levels (Basu et al. 2012) and have described the risk of vasculopathy and increased neonatal morbidity and mortality (Mittendorf et al. 2005, 2006; Jacquemyn et al. 2015), thus retaining questions regarding optimal dosing (Pryde and Mittendorf 2011).

Several hypotheses could be used to explain uncertainties regarding MgSO_4_. First, MgSO_4_ can be prescribed to pregnant women for various indications and, therefore, different physiopathologic contexts, such as neuroprotection of the fetal brain (Doyle et al. 2007), prevention of eclampsia (Ueda et al. 2016), or tocolysis (McNamara et al. 2015). Second, protocols and modalities of administration differ worldwide, and doses of MgSO_4_ administered to the mother and received by the fetus markedly fluctuate. Depending on the MgSO_4_ regimen protocols, total doses administered can vary from 4 g to more than 50 g, particularly when administered for prevention of eclampsia (Pryde and Mittendorf 2011). Third, even if the active component of MgSO_4_ appears to be the Mg^2+^ ion, its large spectrum of action in living cells is a major limitation to decipher mechanisms involved in beneficial and/or adverse effects (Romani 2011). In particular, the Mg^2+^ ion is the physiologic blocker of the glutamate NMDA receptor, which is expressed by neural cells and plays a critical role in the excitotoxic cascade (Desfeux et al. 2010). However, in the developing brain and contrasting to adults, functional NMDA receptors have been recently shown to be highly expressed by endothelial cells from immature microvessels (Henry et al. 2013; Lecointre et al. 2014; Porte et al. 2017).

Consequently, we hypothesized that MgSO_4_ effects leading to neuroprotection and/or vascular adverse effects would result from dose‐related and cell‐specific mechanisms involving the glutamatergic system. Using preclinical and clinical approaches, the objectives of the present study were to perform functional and mechanistic characterization of the effects of clinically relevant doses of MgSO_4_ on neural and vascular cells by targeting neuroprotection, angiogenesis, and hemodynamic parameters and to measure the impact of a 4‐g loading dose of MgSO_4_ on brain hemodynamic parameters in human fetuses by following administration procedures of a validated neuroprotection clinical protocol involving women at imminent risk for preterm delivery.

## Materials and Methods

### Animals

NMRI (National Marine Research Institute) mice (Janvier, Le Genest Saint Isle, France) were kept in a temperature‐controlled room (21 ± 1°C) with an established photoperiod (the lights are on from 7:00 am to 7:00 pm) and with free access to food and tap water. Animal manipulations were performed according to the recommendations of the French Ethical Committee (B7645005) and under the supervision of authorized investigators (B.J.G., authorization no. 7687 from the Ministère de l'Agriculture et de la Pêche).

### Chemicals

5‐Methyl‐10,11‐di‐hydro‐5H‐dibenzo[a,d]cyclohepten‐5,10‐imine maleate (MK801) and isolectin‐B4‐FITC were purchased from Tocris (Bristol, United Kingdom). 7‐aminoactinomycin D (7‐AAD), lactate dehydrogenase (LDH), phenylmethanesulfonyl fluoride (PMSF), phosphatase inhibitor, protease inhibitor cocktails 2 and 3, Hoechst 33258, glutamate, *β*‐nicotinamide adenine dinucleotide (NADH), sodium pyruvate, *β*‐nicotinamide adenine dinucleotide 2′‐phosphate reduced tetrasodium salt hydrate (NADPH), MgSO_4_, and nitro blue tetrazolium were from Sigma Aldrich (Saint‐Quentin Fallavier, France). The ratiometric intracellular calcium probe Fura‐2, pluronic^®^ F‐127 and Cell Tracker green were from Invitrogen (Cergy Pontoise, France). The Apo‐ONE homogeneous caspase‐3/7 kit was provided by Charbonnières‐les‐Bains, France. Isoflurane was from CSF (Cournon, France). Characteristics of the antibodies used in the present study for immunohistofluorescence and Western blot experiments are summarized in Table S1.

### Preparation and treatment of organotypic slices

Brain slices (250 *μ*m) from postnatal day 2 (P2) mouse neonates were cut at 4°C using a vibratome VT1000S Leica (Rueil‐Malmaison, France) as previously described (Henry et al. 2013). Slices were incubated for 6 h at 37°C in artificial cerebrospinal fluid (aCSF) alone (control condition) and in absence or presence of 20 *μ*mol/L MK801, 400 *μ*mol/L glutamate, and (1–8 mmol/L) MgSO_4_. Then, slices were washed with aCSF and metabolic reactions were stopped on ice. Finally, cortical slices were used for immunohistology, Western blot, LDH, and caspase‐3 activity experiments.

### Visualization of necrotic and apoptotic cells

Experiments were conducted in cultured brain slices incubated in the presence of glutamate and/or different concentrations of MgSO_4_. Necrosis is characterized by rapid loss of cytosolic membrane integrity and cell lysis (Maes et al. 2015). Based on these statements, necrotic cell death was visualized using 7‐AAD, a cell‐impermeant fluorescent marker for DNA. Thus, 7‐AAD labeling was detected in cells where the membrane integrity was lost. Red fluorescence was acquired with a Leica DMI 6000B microscope (Rueil‐Malmaison, France). Apoptotic cells were visualized in cultured brain slices using immunohistochemistry targeting the cleaved caspase‐3. 7‐AAD and cleaved caspase‐3 labeling were performed on the same cortical slices to simultaneously visualize the effects of glutamate and MgSO_4_ on cell lysis and apoptosis, respectively.

### Lactate dehydrogenase activity assay

Quantification of LDH activity in the aCSF of cultured cerebral slices was used as an index of cell lysis representative of cellular edema and necrosis (Chan et al. 2013). After the 6 h treatments, 30 *μ*L of aCSF was collected and mixed with 100 *μ*L of a freshly made solution containing 0.5 mmol/L NADH. Conversion of NADH to its oxidized form NAD^+^ was initiated by the addition of 20 *μ*L of 5 mmol/L sodium pyruvate; LDH activity was followed‐up for a period of 15 min by spectrophotometry at 340 nm by measuring the decrease in NADH absorbance with a plate reader (Chameleon; Mustionkatu, Turku, Finland).

### Caspase‐3 activity

Control and treated cortices from brain slices were resuspended in 500 *μ*L of hypotonic lysis buffer and 20 *μ*g of proteins was incubated at 30°C with 100 *μ*L of caspase‐3 buffer containing 1 *μ*L of the caspase‐3 substrate Z‐DEVD‐R110 provided within the Apo‐ONE homogeneous caspase‐3/7 kit from Promega. Fluorescence intensity was quantified every 5 min for 2 h at excitation and emission wavelengths of 485 and 520 nm, respectively, using a plate reader (Chameleon; Mustionkatu).

### Time‐lapse recording of cellular edema

Transverse brain slices from P2 mice were labeled with Cell Tracker green to visualize living cells and cultured under a fluorescent inverted Leica DM microscope under controlled temperatures. Slices were then perfused with aCSF, 400 *μ*mol/L glutamate alone, or in the presence of 1 mmol/L MgSO_4_. Z‐series images of cell tracker green‐positive cells were acquired every 10 min and saved in TIFF format using a computer‐assisted image analysis station (Metamorph; Roper Scientific, Lisses, France). Then, regions of interest (ROIs) corresponding to fluorescent cell bodies present in cortical layers V and VI were integrated to compute cell volume using the Mercator Software (Explora Nova, La Rochelle, France).

### Neuronal and endothelial calcimetry on brain slices

Transverse brain slices from P2 neonates were incubated for 15 min in aCSF with 10 *μ*mol/L of the calcium probe Fura‐2/AM and 0.03% Pluronic^®^ F‐127. Double labeling of the ratiometric calcium probe Fura‐2/AM with (10 *μ*mol/L) isolectin B4‐FITC was used to discriminate between neural and vascular endothelial cells. The fluorescent signals associated with calcium‐bound and calcium‐free Fura‐2 were measured as previously described in both neural and endothelial cells (Jégou et al. 2012). Data were exported to the biostatistic software Prism (GraphPad Inc., San Diego, CA) and two parameters (maximal intensity and area under the curve) were quantified.

### Time‐lapse recording of vascular remodeling and endothelial cell suffering in cortical slices

For imaging of living microvessels, 250‐*μ*m slices from P2 mouse brains and endothelial cells labeled with isolectin‐B4‐FITC were used. Brain slices were cultured for 6 h at a constant temperature (37°C) in a controlled atmosphere (5% CO_2_) under an inverted microscope (Leica) equipped with an incubation chamber (Pecon, Erbach, Germany), computer‐controlled illumination shutters, and filter wheels (Roper Scientific). Slices were continuously perfused with aCSF containing or not containing low (1 mmol/L) and high (6.5 mmol/L) concentrations of MgSO_4_. Images were acquired with ×10 objective every 30 min for 6 h. After acquisition, two parameters, microvessel length and diameters, were quantified using Metamorph software (Jégou et al. 2012). Endothelial cell death was visualized by overlapping 7‐AAD and isolectin‐B4‐FITC acquisitions to discriminate between neuronal and endothelial cell death.

### Immunohistochemistry

After overnight fixation with 4% PFA in PBS, brain slices were rinsed twice with PBS for 10 min and incubated overnight at 4°C with different primary antibodies (Table S1) diluted in the incubation buffer consisting of 1% BSA and 3% Triton X‐100 in PBS. Then, slices were rinsed twice with PBS for 10 min and incubated with the same incubation buffer containing the adequate secondary antibody (Table S1). Fluorescent signals were observed with a Leica DMI 6000B microscope. The specificity of immunoreactions was controlled by replacement of the primary antibodies by PBS.

### In vivo treatment of pregnant mice with MgSO_4_ and quantification of the fetal cortical vasculature

Pregnant mice received a daily subcutaneous injection of sodium chloride (NaCl 9‰) or different doses of MgSO_4_ (100–600 mg/kg) diluted in NaCl from gestational day 15 (GD15) to GD20. The spectrum of doses administered was selected to bracket and compare the low (270 mg/kg; Louzoun‐Kaplan et al. 2008) and high (600 mg/kg; Hoffman et al. 1994) doses of MgSO_4_ used in the literature. These low‐ and high‐injected doses corresponded to a blood peak of magnesium of 1.52 mmol/L and 2.5 mmol/L after 30 min, respectively (Wolf et al. 1991; Hallak et al. 2000). The highest value is consistent with hypermagnesemia values >4.5 mEq/L (2.5 mmol/L) found in some preterm infants (2.5 mmol/L; Ali et al. 2003). Brains were collected at P2 and slices were labeled with isolectin B4‐FITC to visualize the vasculature. For measurement of the vascular network, a ratiometric approach involving Metamorph software (Roper Scientific) was used as previously described (Jégou et al. 2012). For each slice, a threshold was set to distinguish the isolectin B4‐FITC‐positive structures from the background, and the corresponding ROIs were selected by segmentation. For each ROI, the vascular area/cortical area ratio and the thickness of microvessels were calculated. For measurement of the angular position of microvessels, a frame of lines perpendicular to the cortical border for each slice was defined. For microvessels parallel to these lines, Metamorph software arbitrarily attributed an angular value of 0°. The maximal angular value affected was 90°.

### Quantitative real‐time PCR

Generation of polymerase chain reaction (PCR) products was measured in real time by incorporation of the fluorescent dye SYBR Green I using a Bio‐Rad iCycler real‐time PCR machine (Bio‐Rad, Hercules, CA). PCR products were generated using primers described in Table S2.

### Western blot analysis

After in vivo treatment of pregnant mice, cortices from control and MgSO_4_‐treated animals were homogenized in 250 *μ*L of lysis buffer (50 *μ*mol/L HEPES; pH 7.5; 150 mmol/L NaCl; 10 mmol/L EDTA; 10 mmol/L glycerophosphate; 100 mmol/L sodium fluoride; 1% triton ×100; 1 mmol/L PMSF and PI cocktail). After centrifugation of the homogenate (20,000*g*, 15 min), the supernatants were used for Western blotting as previously described (Desfeux et al. 2010). Commercial markers (Seeblue prestained standard; Invitrogen) were used as molecular weight standards.

### In vivo quantification of the fetal cerebral blood flow

Effects of maternal injection of MgSO_4_ on hemodynamic parameters in the fetal brain were analyzed at GD18 using a surgery approach associated with a laser speckle moorFLPI‐2 recording (MOOR Instrument, Axminster, UK). Pregnant mice received a low (100 mg/kg) and high (600 mg/kg) subcutaneous injection of MgSO_4_ and were rapidly anesthetized with isoflurane using an isoflurane Vaporizer (Datex‐Ohmeda; GE Healthcare, Aulnay sous bois, France). Laparotomy was performed to allow access to the uterine horns. The abdominal cavity, especially the exposed uterine horn, was kept moist with warmed physiologic solution. During surgery, the body temperature of the mouse was maintained using a hotplate (Lab‐Line Instruments, Melrose Park, IL). Fetal cerebral blood flow dynamics were measured during 1 min with a pace of 15 pictures acquired per minute. In utero quantification of the fetal cerebral blood flow was performed between 20 and 30 min after the injection of MgSO_4_ to the mother. After measurements, the uterine horn was carefully replaced in the abdominal cavity. The muscles and skin of the surgical wound were sutured separately with sterile Silk Suture Prolene 6‐0, MPP2832H (Ethicon, Lidingö, Sweden).

### Quantification and visualization of Hif‐1*α* after in vivo exposure to MgSO_4_

Thirty min after the low (100 mg/kg) and high (600 mg/kg) subcutaneous injections of MgSO_4_ to GD18 pregnant mice, cortices of the embryos were rapidly removed. For immunohistochemistry, brains were fixed in defrosted PFA 4%, dehydrated by saccharose 30%, and frozen in isopentane immersion (−35°C). Frontal sections (10 *μ*m thick) were cut in a Microm Microtech cryomicrotome HM560 (Brignais, France) and stored in a cryoprotective solution (20% glycerol, 30% ethylene glycol in 0.05 mol/L PBS) at −25°C until use. For Western blot studies, the cortices were rapidly dissected out on ice, immediately frozen on dry ice, and stored at −80°C until homogenization.

### Doppler analysis of fetal cerebral blood flow in human fetuses exposed to 4‐g bolus of MgSO_4_

A prospective, observational cohort was conducted in pregnant women in preterm labor before 33 weeks of gestation admitted to the Department of Obstetrics of Rouen University Hospital. Women with a diagnosis of preeclampsia were not included in the study to restrict patients without pregnancy vascular disease. Patients were enrolled consecutively between July 2014 and November 2015. All patients received MgSO_4_ for fetal neuroprotection according to the local injection protocol: a 4‐g loading dose during 30 min followed by a 1‐g/h maintenance dose during 12 h intravenously. Doppler acquisitions were performed using the ProSound F37 ultrasonographer (Hitachi Aloka Medical America, Inc., Wallingford, CT) attached to a 3.75‐MHz convex transducer. Doppler velocimetry was performed by experienced senior obstetricians just before and after administration of the MgSO_4_ loading dose. Doppler acquisition was similar to that used in the literature to assess fetal anemia (Mari et al. 2000) or the impact of MgSO_4_ on fetal cerebral Doppler flow (Souza et al. 2010). An axial brain section including thalami and cavum septum pellucidi was obtained. The spectral Doppler cursor was placed on the middle cerebral artery within 1 mm of the proximal vessel branch and at least two acquisitions were obtained. The angle between the ultrasound beam and the direction of blood flow were kept as close as possible to 0° to minimize the need for correction. Maximal systole (peak systole velocity; PSV), end diastole, and time‐average maximum velocity were obtained from spectral form. Pulsatility index (PI) and resistance index (RI) were calculated from previous measurements. Comparisons of these parameters before and after the MgSO_4_ loading dose were evaluated. Gestational age, cause of preterm delivery, and Doppler measurements are reported in Table 1. The study was approved by the Local Ethics Committee of Rouen University Hospital (protocol agreement number E2016‐05).

**Table 1:** Doppler indexes and measures of middle cerebral artery of fetuses before and after the administration of a 30‐min loading dose of 4‐g MgSO_4_ in pregnant women

Case no.	Before administration			30 min after							
	PSV	IP	IR	PSV	IP	IR	ΔPSV	ΔIP	ΔIR	Gestational age (WG + days)	Cause of preterm delivery
1	0.596	1.383	0.728	0.525	1.171	0.664	0.071	0.212	0.064	33	Cesarean delivery for fetal ventricular dilatation
2	0.511	2.57	0.93	0.387	1.82	0.84	0.124	0.75	0.09	30	Placenta abruption
3	0.329	4.42	1.13	0.237	2.78	0.805	0.092	1.64	0.325	29 + 1	Preterm labor
4	0.24	1.63	0.76	0.29	1.44	0.75	−0.05	0.19	0.01	26 + 6	Fetal heart rate abnormalities
5	0.21	1.63	0.8	0.34	1.62	0.8	−0.13	0.01	0	26	Fetal heart rate abnormalities
6	0.357	1.29	0.69	0.228	0.65	0.58	0.129	0.64	0.11	26	Fetal heart rate abnormalities
7	0.374	1.12	0.71	0.407	1.31	0.76	−0.033	−0.19	−0.05	32 + 1	Maternal cause: vaso‐occlusive crisis
8	0.432	1.58	0.86	0.324	2.15	1.19	0.108	−0.57	−0.33	32 + 1	Preterm labor
9	0.353	2.42	0.84	0.284	1.61	0.77	0.069	0.81	0.07	30 + 1	Preterm labor, chorioamnionitis
10	0.427	1.47	0.73	0.386	1.58	0.75	0.041	−0.11	−0.02	33	Fetal growth restriction
11	0.425	1.57	0.79	0.421	1.38	0.76	0.004	0.19	0.03	27	Chorioamniotis, fetal heart rate abnormalities
12	0.272	2.5	1.02	0.245	3.24	1.11	0.027	−0.74	−0.09	27 + 5	Preterm labor
13	0.548	2.76	0.92	0.394	1.89	0.83	0.154	0.87	0.09	31 + 5	Preterm labor
14	0.448	1.5	0.75	0.522	1.8	0.82	−0.074	−0.3	−0.07	32	Fetal heart rate abnormalities
15	0.544	1.65	0.8	0.53	1.42	0.76	0.014	0.23	0.04	28 + 6	PPROM, chorioamnionitis
16	0.592	2.15	0.87	0.471	2.88	1	0.121	−0.73	−0.13	32	Preterm labor
17	0.278	1.62	0.82	0.298	1.5	0.77	−0.02	0.12	0.05	26 + 5	Preterm labor
18	0.291	2.05	0.84	0.361	2.46	0.89	−0.07	−0.41	−0.05	26 + 5	Preterm labor
19	0.367	1.87	0.82	0.34	1.31	0.71	0.027	0.56	0.11	27	PPROM, preterm labor
20	0.303	3.65	1.22	0.296	1.94	0.87	0.007	1.71	0.35	28	PPROM, preterm labor
21	0.318	1.79	0.78	0.423	1.37	0.73	−0.105	0.42	0.05	28 + 2	Preterm labor
22	0.425	1.89	0.83	0.444	1.76	0.81	−0.019	0.13	0.02	28 + 2	Preterm labor
23	0.459	1.34	0.71	0.478	1.54	0.77	−0.019	−0.2	−0.06	32 + 1	Fetal growth restriction, fetal heart rate abnormalities
24	0.459	0.81	0.56	0.437	0.97	0.62	0.022	−0.16	−0.06	29	Preterm labor
25	0.432	1.99	0.79	0.436	2.58	1	−0.004	−0.59	−0.21	31 + 5	Chorioamnionitis, preterm labor
26	0.326	0.64	0.47	0.539	0.7	0.47	−0.213	−0.06	0	31 + 1	Fetal growth restriction, fetal heart rate abnormalities
27	0.293	1.36	0.73	0.299	1.44	0.78	−0.006	−0.08	−0.05	25	Preterm labor

PPROM, Preterm premature rupture of membrane.

### Statistical analysis

Statistical tests were performed using the biostatistic software Prism (Graph‐Pad Inc., San Diego, CA). Data are reported as mean ± SEM or sampling distributions. Tests used for each experiment, the number of independent experiments, and *P* values are summarized in Table S3.

## Results

### Effect of MgSO_4_ on cell lysis and intracellular calcium mobilization in nervous cells under excitotoxic conditions

**Figure 1:**
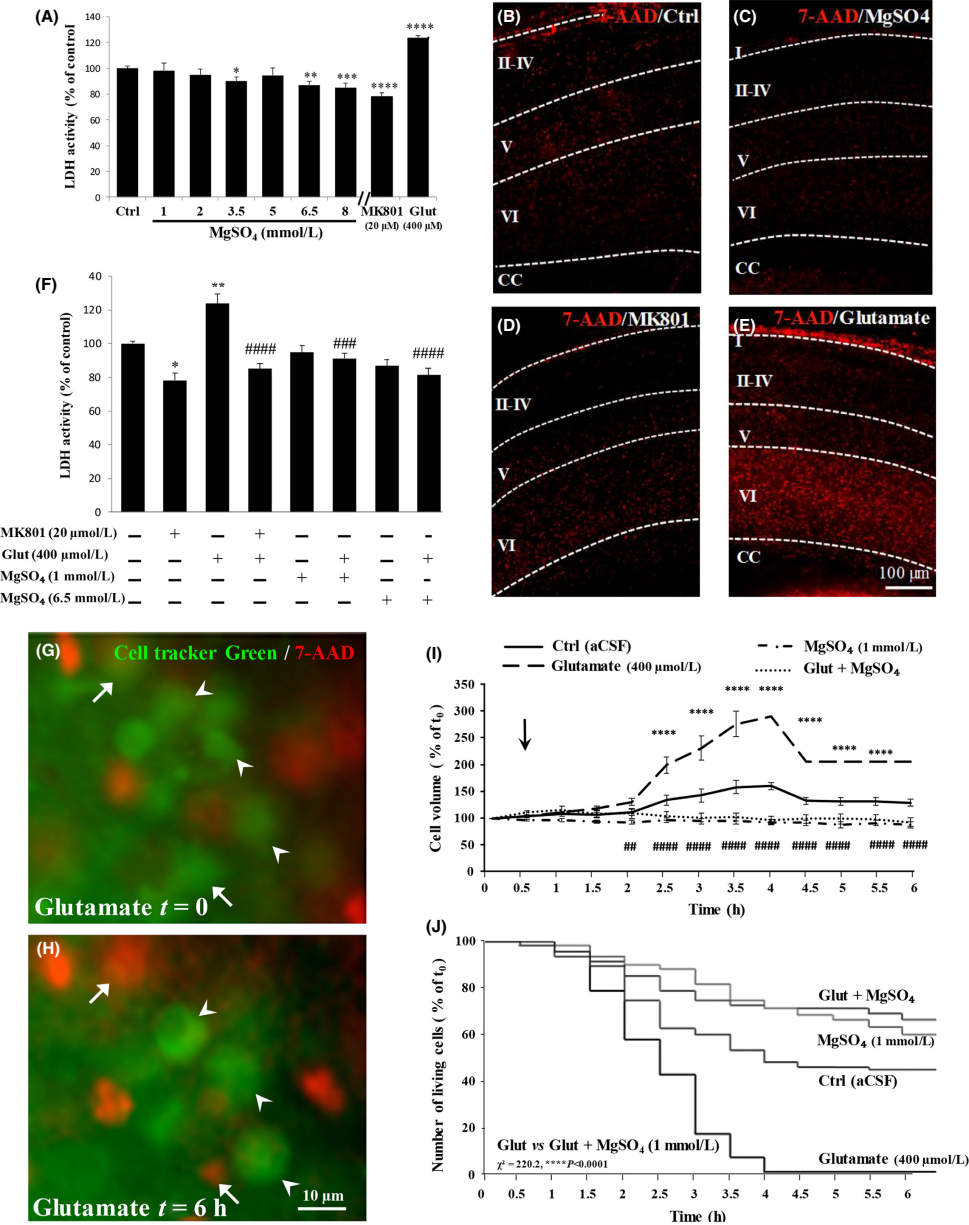
Effects of graded concentrations of MgSO_4_ on excitotoxicity. (A) Quantification of LDH activity on cortical slices from P2 neonates after 6 h of incubation in the absence (Ctrl) or presence of graded concentrations (1–8 mmol/L) of MgSO_4_ alone. The NMDA antagonist MK801 (20 *μ*mol/L) and glutamate (400 *μ*mol/L) were used as negative and positive controls, respectively. One‐way ANOVA test showed a group effect (*F* = 14.14; *P *<* *0.0001), and the Dunnett's multiple comparison test indicated the following: *P <* *0.05; **P <* *0.01; ***P <* *0.001; **** *P *<* *0.0001 versus control. (B–E) Visualization by 7‐AAD labeling of excitotoxic cell death in brain slices from P2 neonates after 6‐h incubation in the absence (B) or presence of 1 mmol/L MgSO_4_ (C), MK801 (20 *μ*mol/L; D), and glutamate (400 *μ*mol/L; E). Note that the pro‐excitotoxic effect of glutamate is preferentially localized in the deep layers. (F) Quantification of LDH activity under excitotoxic conditions. Cortex slices were incubated during 6 h with glutamate (400 *μ*mol/L) in the absence or presence of low (1 mmol/L) and high (6.5 mmol/L) concentrations of MgSO_4_ or MK801 (20 *μ*mol/L). One‐way ANOVA test showed a group effect (*F* = 8.911; *P *<* *0.0001) and the Tukey's multiple comparison test indicated the following: *P <* *0.05; **P <* *0.01 versus control; ###*P *<* *0.001; ####*P *<* *0.0001 versus glutamate. (G,H) Microphotographs from time‐lapse recording visualizing cellular edema in layers V and VI after exposure of cultured cortical slices to glutamate (400 *μ*mol/L). Living cells are labeled with Cell Tracker green and dead cells with 7‐AAD. Arrow heads indicate cells presenting swelling of the cytoplasm and nucleus. Arrows indicate living cells at t0 (G), which were dead (7‐AAD positive) after 6 h (H). (I) Quantification of the cellular edema in cortical layers V and VI from P2 cortical slices exposed for 6 h to aCSF (Ctrl), MgSO_4_ (1 mmol/L), or glutamate (400 *μ*mol/L) alone, or co‐incubated with MgSO_4_ (1 mmol/L). Note that glutamate (400 *μ*mol/L) induces a rapid increase in cell volume representative of cellular edema, which is prevented by MgSO_4_. The arrow indicates the time when drugs were perfused. Two‐way ANOVA test showed a group effect (*F* = 17.19; *P *<* *0.0001) and the Tukey's multiple comparison test indicated the following: ****P <* *0.0001 versus control; ##*P *<* *0.01; ####*P *<* *0.0001 versus glutamate. (J) Survival curves of Cell Tracker green‐positive neurons from layer VI. Cultured slices from P2 neonates were exposed for 6 h to aCSF (Ctrl), MgSO_4_ (1 mmol/L), or glutamate (400 *μ*mol/L) alone, or co‐incubated with MgSO_4_ (1 mmol/L). Log‐rank (Mantel‐Cox) indicated: ****P <* *0.0001 Glut versus Glut.MgSO4 (1 mmol/L).

Quantification of lactate dehydrogenase activity in the culture medium of cultured brain slices is an indicator of loss of cell membrane integrity. Associated with cell edema and cell bursting, it is a good indicator of necrotic cell death (Chan et al. 2013). Six‐h treatment of cultured brain slices from P2 neonates with MgSO_4_ alone in concentrations ranging from 1 to 8 mmol/L induced a concentration‐dependent reduction of LDH activity (Fig. 1A). The same effect was quantified with MK801 (*P* < 0.0001; 20 *μ*mol/L), whereas glutamate (400 *μ*mol/L), which was used as a positive control of excitotoxicity, induced a strong increase in LDH activity (*P* < 0.0001; Fig. 1A). Visualization of cells with altered membrane integrity was performed using 7‐AAD labeling (Fig. 1B–E). Whereas in control, MK801 (20 *μ*mol/L), and MgSO_4_ (1 mmol/L) conditions, the dotted 7‐AAD labeling was weak (Fig. 1B–D), the treatment of brain slices with glutamate (400 *μ*mol/L) resulted in a strong increase in the fluorescence intensity which was obvious in deep cortical layers V and VI of the P2 neocortex (Fig. 1E). When MgSO_4_ and glutamate (400 *μ*mol/L) were co‐incubated, both low (1 mmol/L; *P *<* *0.001) and high (6.5 mmol/L; *P *<* *0.0001) concentrations of MgSO_4_ prevented the effect of glutamate on LDH activity, indicating potent anti‐excitotoxic action of MgSO_4_ (Fig. 1F). Consistent with these data, MK801 (20 *μ*mol/L) mimicked the effect of MgSO_4_ on glutamate‐induced LDH activity (*P *<* *0.0001; Fig. 1F).

In addition to membrane disruption, cell edema is another criterion of excitotoxicity. Cell swelling results from a massive entry of water into the cells and overstimulation of the ionotropic glutamate receptors AMPA/Kainate and NMDA (Ingvar et al. 1988). Because the anti‐excitotoxic effect of MgSO_4_ was similar between 1 mmol/L and 6.5 mmol/L concentrations (Fig. 1F), we performed time‐lapse experiments on cultured brain slices to further characterize the effects of the 1 mmol/L concentration of MgSO_4_ on cell swelling. In deep cortical layers V and VI, treatment of cultured brain slices with glutamate (400 *μ*mol/L) resulted in a rapid increase in the volume of cortical neurons previously labeled with the Cell Tracker green probe (Fig. 1G–I). Several cells presenting a typical fusiform shape became progressively round (Fig. 1H; arrowheads). Morphometric analysis revealed a significant effect of glutamate (400 *μ*mol/L) on cell volume 2 h after the beginning of treatment (*P *<* *0.0001; Fig. 11). With longer incubation periods with glutamate (400 *μ*mol/L), cell swelling resulted in cell burst and cell death revealed by 7‐AAD labeling (Fig. 1H; arrows). As found for LDH activity, the low (1 mmol/L) concentration of MgSO_4_ abrogated the effects of glutamate on both cell swelling (Fig. 1I) and cell death (*P *<* *0.0001; Fig. 1J). In addition to excitotoxic cell death, we also analyzed several indicators of apoptosis (caspase‐3 cleavage, caspase activity, Bax expression) and found no significant effect of MgSO_4_ (Fig. S1).

**Figure 2:**
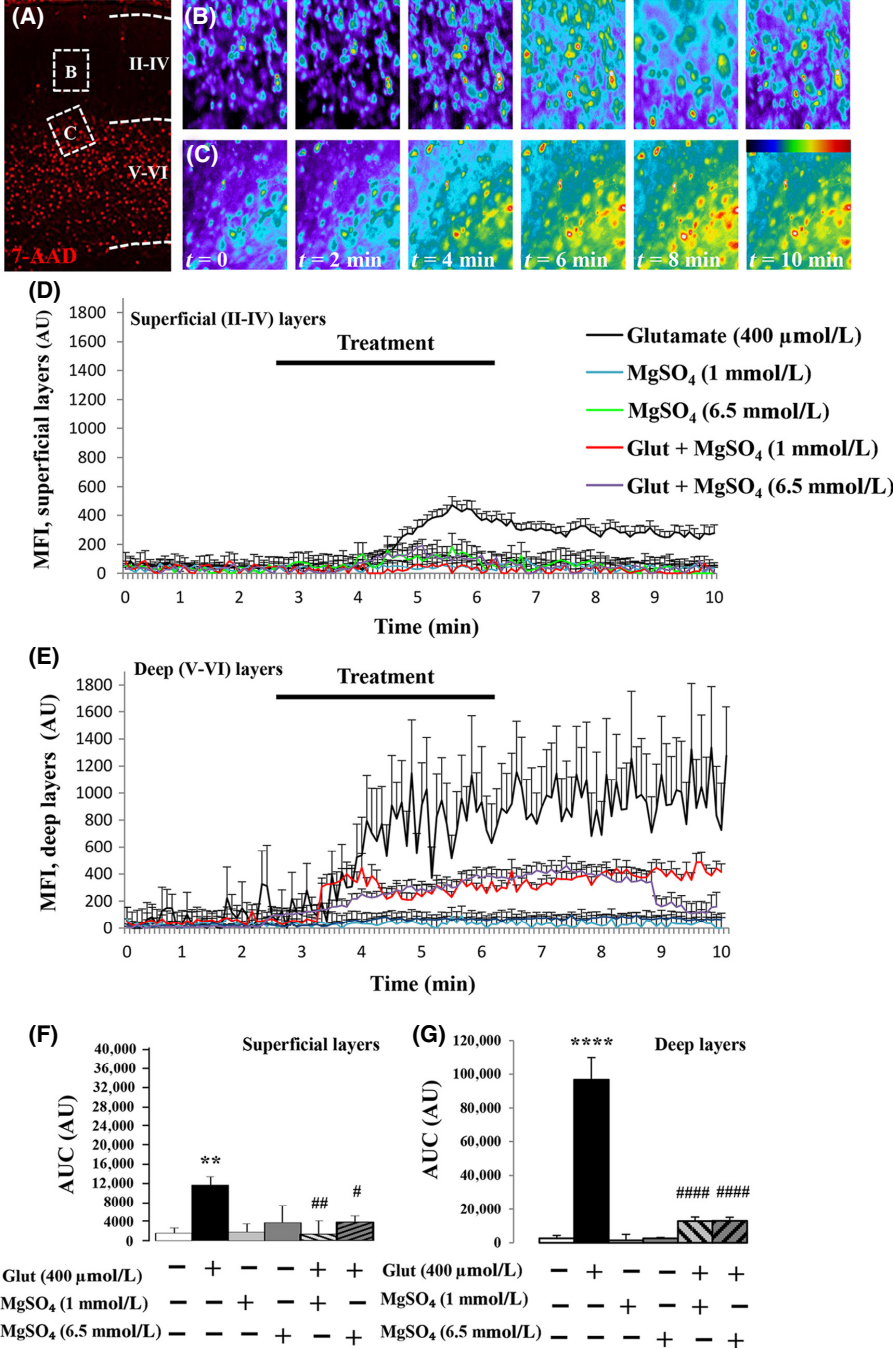
Effects of MgSO_4_ on intracellular calcium levels under basal and excitotoxic conditions. (A) Visualization at low magnification of the excitotoxic cell death (7‐AAD labeling) induced by 400 *μ* glutamate. Dead cells are mainly localized in deep cortical layers V and VI. The inserts indicate the fields recorded for calcimetry experiments in the superficial (B) and deep (C) cortical layers. (B, C) Microphotographs visualizing the kinetics of intracellular calcium mobilization in cells exposed to glutamate (400 *μ*mol/L) in superficial (B) and deep (C) cortical layers. The color bar indicates low (blue) and high (red) levels of calcium. (D) Mean kinetics of calcium mobilization in cells from the superficial layers. Calcimetry was performed after incubation of P2 cortical slices with the ratiometric calcium probe Fura‐2. In basal conditions, slices were perfused with aCSF. After 3 min, aCSF was replaced by different bathing solutions consisting of glutamate (400 *μ*mol/L), MgSO_4_ (1 mmol/L), and MgSO_4_ (6.5 mmol/L), alone or in association. (E) Mean kinetics of calcium mobilization in cells from the deep cortical layers. In basal condition, slices were perfused with aCSF. After 3 min, aCSF was replaced by different bathing solutions consisting of glutamate (400 *μ*mol/L), MgSO_4_ (1 mmol/L), and MgSO_4_ (6.5 mmol/L), alone or in association. (F) Statistical analysis of the mean areas under the curve quantified for each tested condition in the superficial layers. One‐way ANOVA test (*F* = 3.251; *P *=* *0.0088) followed by Dunnett's multiple comparison test **P <* *0.01 versus control; #*P *<* *0.05; ##*P *<* *0.01 versus glutamate. (G) Statistical analysis of the mean areas under the curve quantified for each tested condition in the deep layers. One‐way ANOVA test (*F* = 47.24; *P *<* *0.0001) and Dunnett's multiple comparison test indicated the following: ****P <* *0.0001 versus control; ####*P *<* *0.0001 versus glutamate.

Glutamate‐induced excitotoxicity is associated with a massive increase in intracellular calcium levels (Stanika et al. 2009). Thus, to further characterize the neuroprotective effect of MgSO_4_, we investigated its effects on glutamate‐induced calcium mobilization in P2 cortical slices (Fig. 2). Calcium microfluorimetry experiments revealed two patterns of calcium response after incubation with 400 *μ*mol/L glutamate (Fig. 2A–C). In superficial cortical layers, where glutamate is not excitotoxic at this developmental stage, treatment of slices with the neurotransmitter induced a moderate increase in intracellular calcium levels (Fig. 2B and D). When low (1 mmol/L) and high (6.5 mmol/L) concentrations of MgSO_4_ were co‐incubated, they abrogated the effect of glutamate on calcium mobilization (Fig. 2D). In deep cortical layers, where glutamate is excitotoxic, treatment of slices with the neurotransmitter induced a massive increase in intracellular calcium levels that remained sustained even after washing (Fig. 2C and E). The co‐incubation of glutamate with low (1 mmol/L) and high (6.5 mmol/L) concentrations of MgSO_4_ strongly reduced the calcium mobilization (Fig. 2E). Quantification of the areas under the curves revealed that glutamate was much more potent to promote calcium flux in deep layers than in superficial layers, and that low and high concentrations of MgSO_4_ were equally potent to prevent the glutamate‐induced calcium mobilization in deep layers (*P *<* *0.0001; Fig. 2F and G). Altogether, these data indicate for the first time that a large range of MgSO_4_ concentrations exerts similar neuroprotective effects against glutamate‐induced excitotoxicity.

### Low and high neuroprotective concentrations of MgSO_4_ exert differential effects on endothelial cell activity

**Figure 3:**
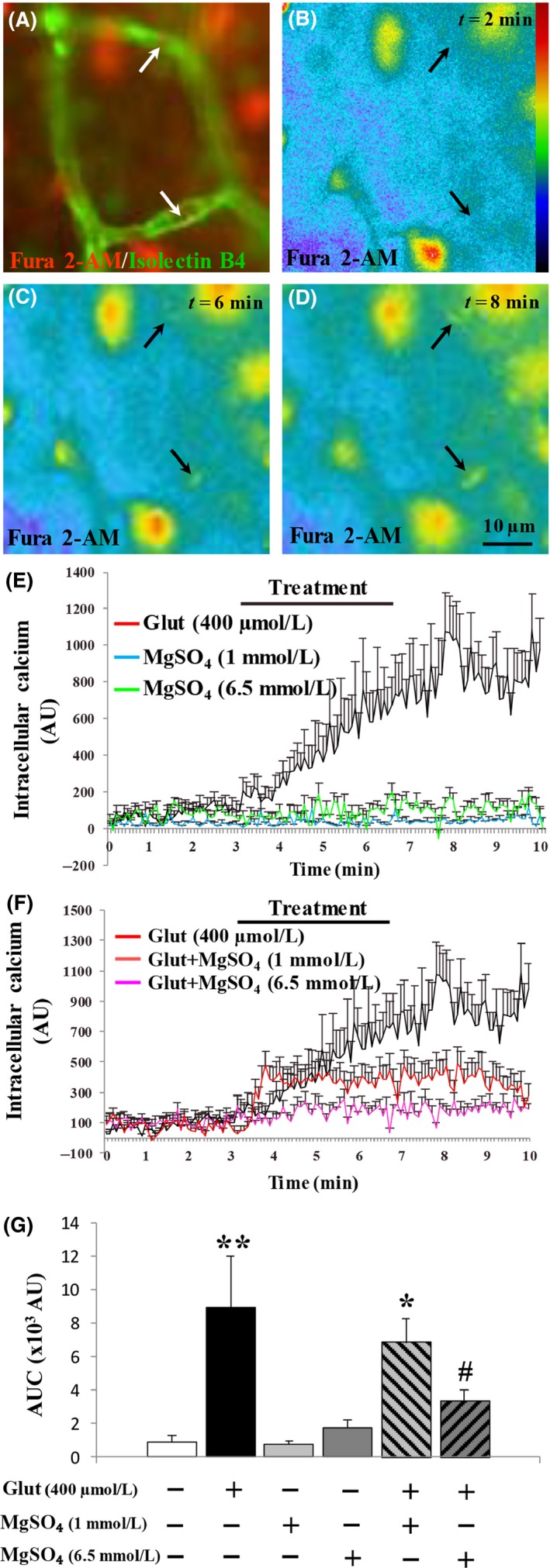
Effects of MgSO_4_ on intracellular calcium mobilization in endothelial cells from P2 neonate cortices. (A) Microphotographs visualizing endothelial cells labeled with isolectin B4‐FITC and loaded with the calcium probe Fura‐2/AM (arrow). (B‐D) False color kinetics of the 340 nm/380 nm ratio representative of intracellular calcium mobilization in cortical endothelial cells exposed to glutamate (400 *μ*mol/L; arrows). The color bar is indicative of low (blue) and high (red) intracellular levels of calcium. (E) Mean kinetics of calcium mobilization in endothelial cells exposed to low (1 mmol/L) and high (6.5 mmol/L) concentrations of MgSO_4_ and glutamate (400 *μ*mol/L) alone. (F) Mean kinetics of calcium mobilization in endothelial cells exposed to low (1 mmol/L) and high (6.5 mmol/L) concentrations of MgSO_4_ in association with glutamate (400 *μ*mol/L). (G) Statistical analysis of the mean areas under the curve quantified for each tested condition. One‐way ANOVA test showed a group effect (*F* = 5.54; *P *=* *0.0016) and Dunnett's multiple comparison test indicated the following: *P <* *0.05; **P <* *0.01 versus control and #*P *<* *0.05 versus glutamate.

Several recent studies demonstrated the expression of functional glutamatergic (NMDA) receptors by neonatal endothelial cells from brain microvessels (Henry et al. 2013; Lecointre et al. 2014), suggesting that MgSO_4_ would impact their activity. Thus, we investigated the effects of MgSO_4_ on calcium mobilization in brain microvessels by fluorimetry. Isolectin B4‐FITC was used to co‐label endothelial cells previously loaded with the calcium probe Fura 2‐AM (Fig. 3A). False color representation (Fig. 3B‐D; arrows) and cell quantification (Fig. 3E) showed an increase in the 340/380 nm ratio in endothelial cells of brain slices perfused with glutamate (400 *μ*mol/L). When MgSO_4_ was applied alone at low (1 mmol/L) and high (6.5 mmol/L) concentrations on brain slices, no effect was found regarding calcium mobilization (Fig. 3 E and G). When it was co‐incubated, MgSO_4_ inhibited the stimulatory effect of glutamate. However, in contrast to nervous cells (Fig. 2 E and G), the effect of MgSO_4_ was concentration‐dependent and significant only for the high (6.5 mmol/L) concentration of MgSO_4_ (*P *<* *0.05; Fig. 3F and G). Altogether, these data indicate that two concentrations of MgSO_4_ with similar neuroprotective actions exert dose‐dependent effects on the endothelial cell activity and would be able to impact the vascular plasticity.

### Low and high neuroprotective concentrations of MgSO_4_ differently impact vascular suffering and endothelial cell death

**Figure 4:**
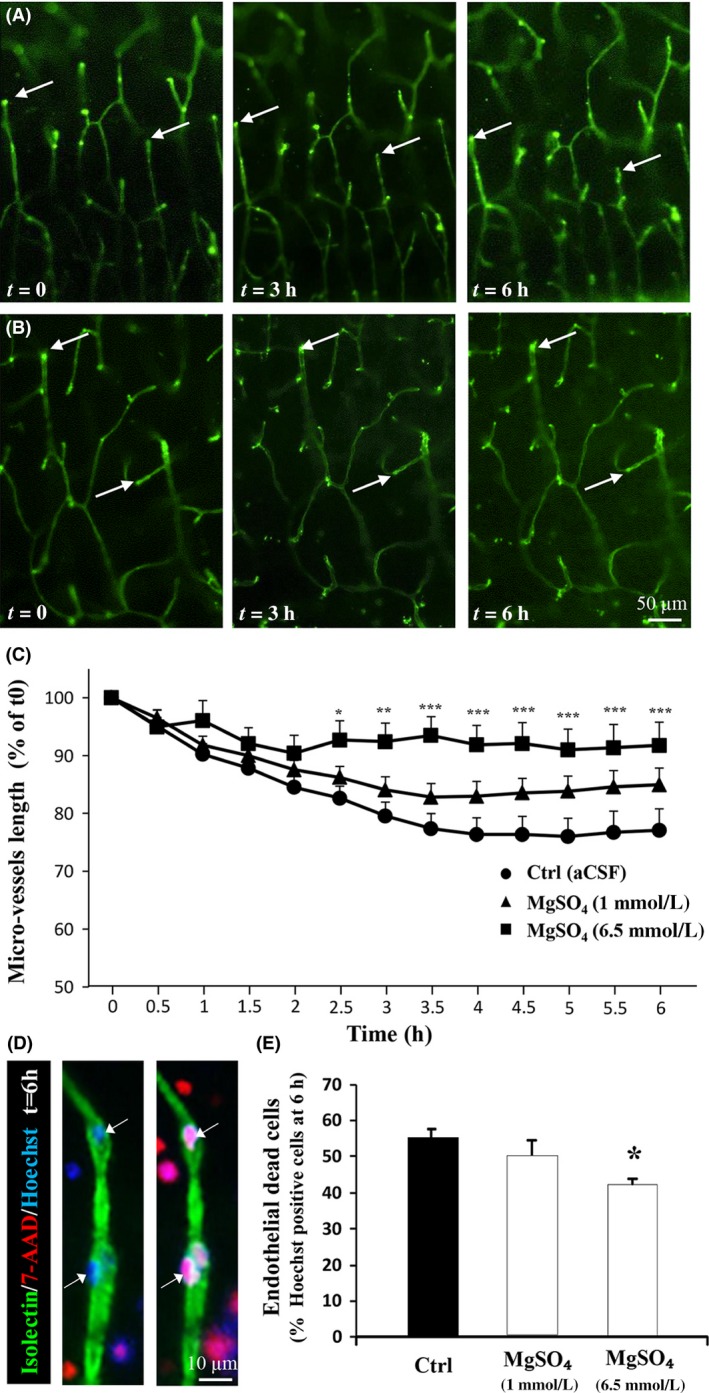
Effects of MgSO_4_ on vascular plasticity and endothelial cell death in cultured cortical slices. (A, B) Time‐lapse acquisition of cortical microvessels labeled with isolectin‐B4‐FITC in control conditions (A) and in a high (6.5 mmol/L) concentration of MgSO_4_ (B). Note that in control conditions, brain microvessels progressively retract within the slice, a criterion of vascular suffering. This vascular suffering is prevented by MgSO_4_. (C) Quantification of the vascular retraction observed in the cortex of neonatal brain slices cultured under control (aCSF), low (1 mmol/L), and high (6.5 mmol/L) concentrations of MgSO_4_. Two‐way ANOVA test showed a group effect (*F* = 1.343; *P *=* *0.1662) and Tukey's multiple comparison test indicated the following: *P <* *0.05; **P <* *0.01; ***P <* *0.001 versus control. (D) High magnification to visualize triple fluorescent labeling of microvessels (isolectine B4‐FITC), dead cells (7‐AAD), and nuclei (Hœchst) in the cortex from P2 brain slices cultured in control conditions during two incubation periods. Note that cell death occurred in neural cells and also in endothelial cells (arrows). (E) Quantification of the effects of low (1 mmol/L) and high (6.5 mmol/L) concentrations of MgSO_4_ on endothelial cell death. One‐way ANOVA test showed a group effect (*F* = 3.242; *P *=* *0.0547) and Tukey's multiple comparison test indicated *P <* *0.05 versus control.

Time‐lapse tracking is a potent approach to follow the suffering of cortical microvessels in cultured neonatal brain slices (Jégou et al. 2012). In basal conditions, a 6‐h recording period showed that several cortical microvessels progressively retracted within the slice (Fig. 4A; arrows). In contrast, in the presence of a high (6.5 mmol/L) concentration of MgSO_4_, this effect was markedly reduced (Fig. 4B; arrows). Time course quantification of brain microvessel length showed that, in the control condition, the vessel retraction was approximately 20% after 4 h and reached a plateau from 4 to 6 h (Fig. 4C). MgSO_4_ induced a concentration‐dependent inhibition of the vascular suffering, which was significantly reduced only for the high concentration (6.5 mmol/L; Fig. 4C; *P *<* *0.05; *P *<* *0.01; *P *<* *0.001). Triple fluorescent labeling with isolectin‐B4‐FITC, 7‐AAD, and Hœchst revealed that vascular retraction was associated with endothelial cell death (Fig. 4D; arrows). Quantification of 7‐AAD‐positive nuclei indicated that only the high concentration of MgSO_4_ reduced death of immature endothelial cells (Fig. 4E; *P *<* *0.05). These data give the first indication of a concentration‐dependent action of MgSO_4_ on microvessel plasticity and endothelial cell death in cultured brain slices from mouse neonates, and support in vivo impairments of brain angiogenesis.

### Low and high neuroprotective doses of MgSO_4_ differently alter brain angiogenesis in vivo

**Figure 5:**
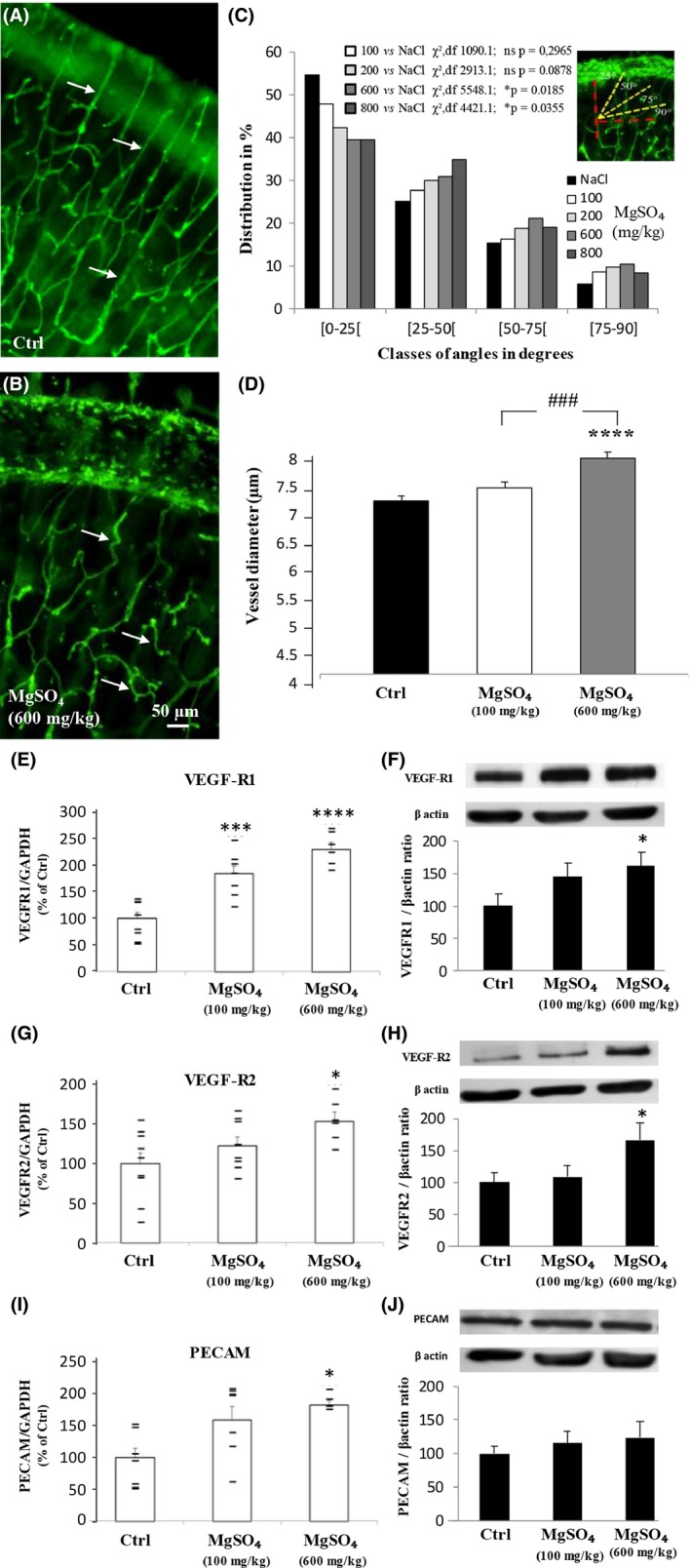
In vivo effects of in utero MgSO_4_ exposure on cortical microvasculature of P2 neonates and on the expression of cortical pro‐angiogenic factors. (A, B) Effects of treatment of pregnant mice with 0.9% NaCl (A) and MgSO_4_ 600 mg/kg (B) from GD15 to GD20 on the cortical microvasculature of P2 neonates. Note the preferential radial orientation of the cortical microvessels in the control group (A; arrows) and the predominance of tortuous vessels in treated animals (B; arrows). (C) Dose‐dependent effect of MgSO_4_ on the distribution of cortical microvessels by class of angles in the neocortex of P2 neonates. Statistical analysis was performed using the *χ* ^2^ test. (D) Effect of in utero exposure to low (100 mg/kg) and high (600 mg/kg) concentrations of MgSO_4_ on the mean diameter of cortical microvessels. One‐way ANOVA test showed a group effect (*F* = 13.20; *P *<* *0.0001) and Tukey's multiple comparison test indicated the following: ****P <* *0.0001 versus control and ###*P *<* *0.001 versus MgSO_4_ (600 mg/kg). (E, F) Quantification of VEGF‐R1 mRNA (E) and protein (F) levels in the neocortex from P2 pups whose mothers were exposed to NaCl (0.9%; Ctrl) or low (100 mg/kg) and high (600 mg/kg) doses of MgSO_4_ from GD15 to GD20. (E) One‐way ANOVA test showed a group effect (*F* = 24.95; *P *<* *0.0001) and Tukey's multiple comparison test indicated the following: ***P <* *0.001; ****P <* *0.0001 versus control. (F) One‐way ANOVA test showed a group effect (*F* = 3.707; *P *=* *0.0329) and Tukey's multiple comparison test indicated *P <* *0.05 versus control. (G, H) Quantification of VEGF‐R2 mRNA (G) and protein (H) levels in the neocortex from P2 pups whose mothers were exposed to NaCl (0.9%; Ctrl) or low (100 mg/kg) and high (600 mg/kg) doses of MgSO_4_ from GD15 to GD20. (G) One‐way ANOVA test showed a group effect (*F* = 5.072; *P *=* *0.0165); Tukey's multiple comparison test indicated *P <* *0.05 versus control. (H) One‐way ANOVA test showed a group effect (*F* = 3.539; *P *=* *0.038) and Tukey's multiple comparison test indicated *P <* *0.05 versus control. (I, J) Quantification of PECAM/CD31 mRNA (I) and protein (J) levels in the neocortex from P2 pups whose mothers were exposed to NaCl (0.9%; Ctrl) or low (100 mg/kg) and high (600 mg/kg) doses of MgSO_4_ from GD15 to GD20. (I) One‐way ANOVA test showed a group effect (*F* = 4.348; *P *=* *0.0288); Tukey's multiple comparison test indicated *P <* *0.05 versus control. (J) Tukey's multiple comparison test indicated no significance versus control (*F* = 0.3927; *P *=* *0.6795).

Pregnant mice were exposed to graded doses of MgSO_4_ from GD15 to GD20, a period of intense angiogenesis and vascular‐dependent neuronal migration in the developing cortex (Won et al. 2013). Visualization of the microvascular network in the cortex of P2 neonates revealed that in control animals, microvessels present a preferential radial organization (Fig. 5A; arrows). In contrast, after in utero exposure to a high dose of MgSO_4_ (600 mg/kg), brain microvessels were disorganized and tortuous (Fig. 5B; arrows). Quantification of the angular orientation of the microvessels in the neocortex of newborns revealed a dose‐dependent effect of MgSO_4_ comprising a progressive disorganization of blood vessels (Fig. 5C). In addition, MgSO_4_ induced a significant and dose‐dependent increase in the diameter of the microvessels (*P *<* *0.0001; Fig. 5D). These data constitute the first preclinical demonstration that in vivo MgSO_4_ administration during fetal life exerts dose‐dependent impairments on the cortical vasculature, suggesting an action of MgSO_4_ on the expression of angiogenic pathways.

Quantitative RT‐PCR experiments revealed that in vivo treatment of pregnant mice with low (100 mg/kg) and high (600 mg/kg) doses of MgSO_4_ induced a significant increase in VEGF‐R1 mRNA levels (Fig. 5E). At the protein level, Western blot experiments indicated that VEGF‐R1 expression was significantly (*P *<* *0.05) increased in the neocortex of animals exposed to the high dose of MgSO_4_ (Fig. 5F). Regarding VEGF‐R2, in vivo MgSO_4_ treatment induced a dose‐dependent (*P *<* *0.05) increase in mRNA levels (Fig. 5G). However, in contrast to VEGF‐R1, only the high dose of MgSO_4_ was efficient (*P *<* *0.05; Fig. 5G). Similarly, a strong increase in VEGF‐R2 protein levels was found for the high dose of MgSO_4_ tested (*P *<* *0.05; Fig. 5H). Quantification of the endothelial cell marker PECAM/CD31 revealed a significant increase in mRNA levels for the high dose of MgSO_4_, which was not found at the protein level (Fig. 5I and J). These data constitute the first demonstration that the treatment of pregnant mice with high doses of MgSO_4_ impairs in a dose‐dependent manner the expression of VEGF‐R1 and VEGF‐R2 in the cortex of mouse neonates.

### Low and high neuroprotective doses of MgSO_4_ differently affect fetal brain perfusion and Hif‐1*α* stabilization in vivo

**Figure 6:**
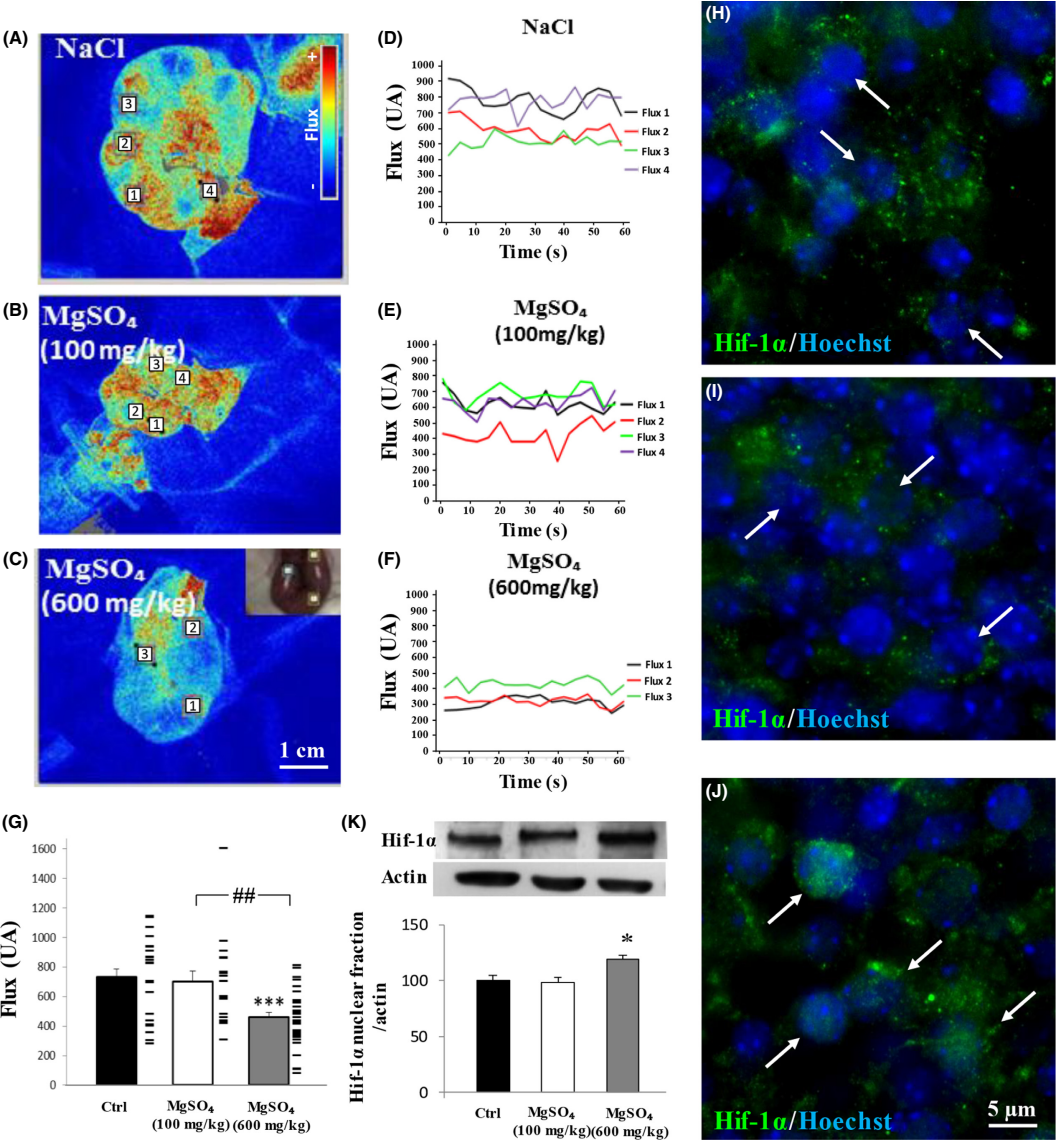
Effects of in utero MgSO_4_ exposure on brain perfusion of fetuses and on Hif‐1*α* expression. (A–C) False color visualization by laser speckle imagery of blood–brain perfusion in fetuses whose mothers were exposed to NaCl (0.9%; A) or low (100 mg/kg; B) and high (600 mg/kg; C) doses of MgSO_4_. The color bar is indicative of low (blue) and high (red) cerebral blood perfusion. Numbers indicate the fetal heads selected for the quantification of cerebral blood perfusion. (D–F) 1‐min in utero recording of the cerebral blood flux from fetuses whose mother were injected 30 min earlier with NaCl (0.9%; A) or low (100 mg/kg; B) and high (600 mg/kg; C) doses of MgSO_4_. For each condition, the curve numbers correspond to the fetuses identified in A–C. (G) One‐way ANOVA analysis showed a group effect (*F* = 8.469; *P *=* *0.0005) and Tukey's multiple comparison test indicated the following: ***P <* *0.001 versus control; ##*P *<* *0.01. (H–J) Visualization by immunohistochemistry of Hif‐1*α* labeling in cortical neurons of fetuses 30 min after treatment of the GD18 pregnant mice with NaCl (0.9%; H) or low (100 mg/kg; I) and high (600 mg/kg, J) doses of MgSO_4_. Note that in control conditions, Hif‐1*α* immunoreactivity is cytosolic (H), whereas nuclear translocation occurred (J) for the highest dose of MgSO_4_. (K) Quantification by Western blot of Hif‐1*α* levels in the nuclear fraction of cortices from GD20 fetuses exposed in utero to NaCl (0.9%) or low (100 mg/kg) and high (600 mg/kg) concentrations of MgSO_4_. One‐way ANOVA analysis showed a group effect (*F* = 6.212; *P *=* *0.0345) and Tukey's multiple comparison test indicated the following: *P <* *0.05 versus control; #*P *<* *0.05 versus MgSO_4 _100 mg/kg.

A mechanistic link has been established between increased VEGF‐R2 expression and hypoxia (Marti et al. 2000). To determine whether MgSO_4_ would induce hemodynamic impairments in the brain of fetuses, we performed in utero laser speckle recordings in GD18 pregnant mice exposed to low and high doses of MgSO_4_ (Fig. 6). In vivo blood flow imaging of fetuses in uterine horns was performed under control conditions (Fig. 6A) and 30 min after the administration of low (100 mg/kg; Fig. 6B) and high (600 mg/kg; Fig. 6C) doses of MgSO_4_. With the high dose of MgSO_4_, false color images revealed a strong and global decrease in the fetal perfusion (Fig. 6C). Quantification of blood perfusion within fetal heads (Fig. 6A–C; squares) showed a significant reduction of blood flow in brain fetuses exposed to the high dose of MgSO_4_ (*P *<* *0.001; Fig. 6D–G). Immunohistochemistry experiments targeting Hif‐1*α* showed that in the cortex of fetuses whose mothers were treated with 0.9% NaCl (Fig. 6H) or 100 mg/kg MgSO_4_ (Fig. 6I), Hif‐1*α* immunolabeling was mainly cytosolic and poorly co‐localized with nuclei (arrows; Fig. 6H and I). In contrast, in the brain of fetuses from pregnant mice exposed to the high (600 mg/kg) dose of MgSO_4_, Hif‐1*α* immunolabeling co‐localized with nuclei in several nervous cells (Fig. 6J). Quantification by Western blot of the nuclear fraction of Hif‐1*α* in cortex extracts revealed a significant effect of the high (600 mg/kg) dose of MgSO_4_ (*P *<* *0.05; Fig. 6K). These data indicate for the first time that in vivo treatment of pregnant mice with a high dose of MgSO_4_ results in brain hypoperfusion and Hif‐1*α* stabilization in nervous cells.

### A low (4‐g) loading dose of MgSO_4_ does not impair hemodynamic indicators in the human fetal brain

**Figure 7:**
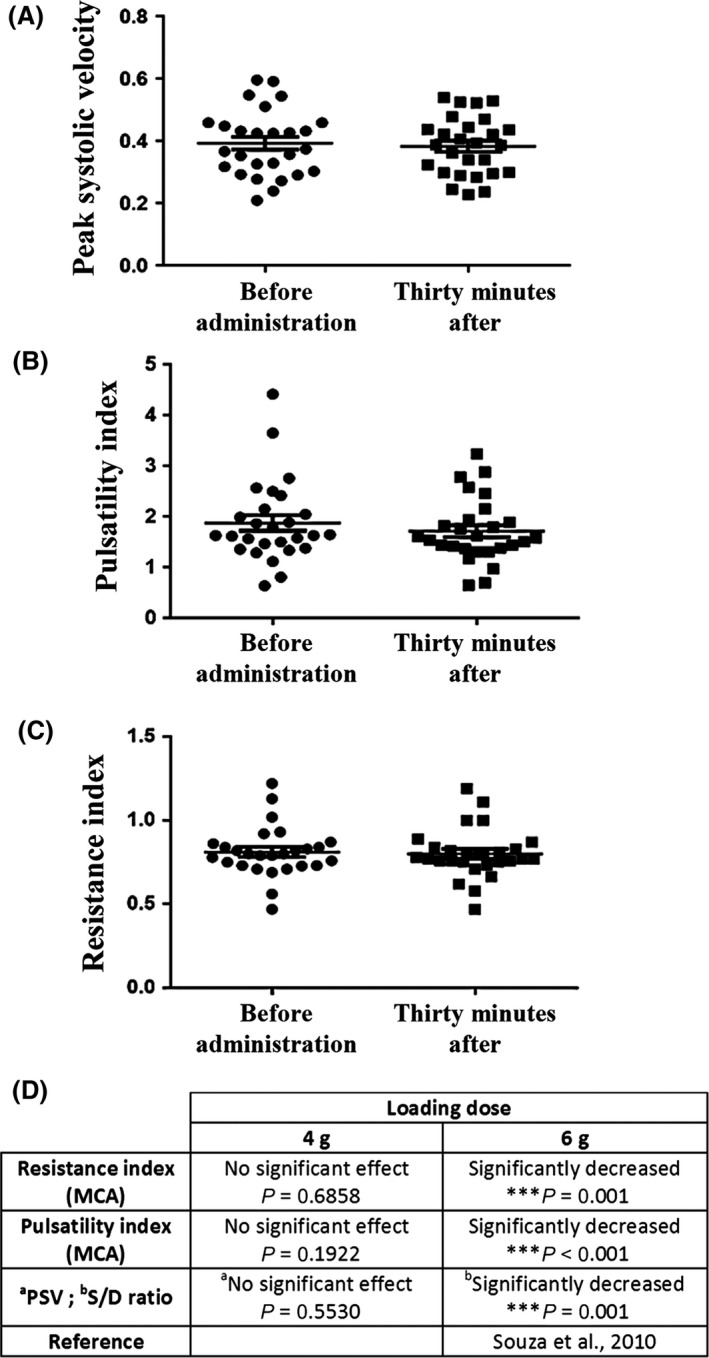
Effects of MgSO_4_ administration in pregnant women on Doppler flow parameters of the fetal brain. (A–C) Pregnant women with gestational ages ranging from 26 to 33 weeks received an intravenous bolus of 4‐g MgSO_4_. The quantity and the mode of administration of MgSO_4_ were those defined by protocols which are routinely used in clinics for fetal neuroprotection (Crowther et al. 2003; Marret et al. 2007). Peak systolic velocity (A), pulsatility index (B), and resistance index (C) were measured in the middle cerebral artery of fetuses before and 30 min after the administration of MgSO_4_ to the pregnant women. Statistical analysis revealed no significant differences using the paired *t* test. (D) Comparison of Doppler flow parameters acquired in the fetal brain after 4 g or 6 g loading doses of MgSO_4_.

Previous studies from Souza and co‐workers investigated in humans the effect of MgSO_4_ on fetal cerebral blood flow in preeclampsia (Souza et al. 2010). Results revealed that after a 6‐g MgSO_4_ loading dose, resistance index (RI) and pulsatility index (PI) significantly decreased in the fetal middle cerebral artery. We performed a similar follow‐up of hemodynamic criteria in the middle cerebral artery of fetuses exposed to a bolus of 4‐g MgSO_4,_ a dose that is in accordance with currently used and validated neuroprotection protocols (Crowther et al. 2003; Marret et al. 2007). In all. 27 human fetuses were included in the study (Table 1). Mean gestational age when Doppler velocimetry was performed was 29.3 ± 0.5 weeks of gestation (WG). Contrasting to results obtained with a 6‐g loading dose, data analysis revealed no statistically significant reduction in the mean RI, PI, and PSV (Fig. 7A–D). These results demonstrate that intravenous infusion of 4‐g MgSO_4_ in pregnant women does not impair hemodynamic indicators in the middle cerebral artery of fetuses.

## Discussion

Currently, MgSO_4_ is used in obstetrics departments for several indications, that is, preeclampsia (Ueda et al. 2016), tocolysis (McNamara et al. 2015), and neuroprotection (Doyle et al. 2007). However, modalities and doses of MgSO_4_ administered to pregnant women strongly vary worldwide. Some case reports showed adverse effects, including brain vasculopathies on fetuses from women who received cumulative high doses of MgSO_4_ (Mittendorf et al. 2006; Abbassi‐Ghanavati et al. 2012; De Jesus et al. 2015). For these reasons, despite that the use of MgSO_4_ is recommended for neuroprotection by several colleges of obstetricians and gynecologists, no consensus exists regarding the optimal regimen of MgSO_4_ (McPherson et al. 2014). These uncertainties result from the fact that mechanisms leading to neuroprotection and/or vascular side effects of MgSO_4_ in the developing brain are not understood or controversial (Hoffman et al. 1994; Galinsky et al. 2016). Based on these statements, using preclinical models and clinical approaches, we performed molecular and mechanistic characterization of the effects of graded doses of MgSO_4_ on several physiologic and pathophysiologic processes occurring in the developing brain of fetuses and neonates by focusing on excitotoxicity, apoptosis, and angiogenesis. In addition, we measured, in human fetuses, hemodynamic action of a 4‐g bolus of MgSO_4_, a loading dose already validated and currently used in neuroprotection clinical protocols (Crowther et al. 2003; Marret et al. 2007), to compare it to higher loading doses used in preeclampsia protocols, and which have been shown to induce hemodynamic perturbations in the fetal brain (Souza et al. 2010; Farshchian et al. 2012).

In preterm and term neonates, excitotoxicity represents a major process leading to brain lesions (Volpe 2009), and the biologic plausibility for the neuroprotective role of MgSO_4_ would result, at least in part, from anti‐excitotoxic action (Clerc et al. 2013). Thus, we investigated the effects of graded **concentrations** of MgSO_4_ on excitotoxic cell death by focusing on cellular edema, cell burst, and LDH activity. Experiments revealed that MgSO_4_ markedly prevented glutamate‐induced excitotoxicity and that the anti‐excitotoxic effect of MgSO_4_ was similar between the lowest (1 mmol/L) and the highest (6 mmol/L) neuroprotective concentrations tested. The lack of difference in terms of neuroprotection between the two concentrations resulted from a maximum effect or “plateau” supporting that, when neuroprotection is effective, increasing MgSO_4_ levels would not improve beneficial effects. Moreover, it has been shown that, in the developing brain, blockade of the NMDA receptor would be pro‐apoptotic (Hansen et al. 2004; Aligny et al. 2014). These data raised the question of pro‐apoptotic side effects of MgSO_4_. Using cultured brain slices from mouse neonates, our results revealed that MgSO_4_ is not pro‐apoptotic on its own and no significant effect of MgSO_4_ was found on caspase‐3 activity, caspase‐3 cleavage, or Bax expression for all the concentrations tested, whereas a positive control, MK801, did. These differential effects of MgSO_4_ and MK801 or ketamine on apoptosis could be explained by specific pharmacological properties. On depolarization, the Mg^2+^ block of all NMDA receptor subtypes is relieved (Huang and Gibb 2014); in contrast, the blocking effect of ketamine and MK801 on the NMDA receptor is much longer (Jin et al. 2013).

Several clinical studies suggested that in utero exposure to MgSO_4_ would impact the vascular system and hemodynamic parameters of the fetus (Mittendorf et al. 2006; De Jesus et al. 2015). Consistent with these clinical observations, MgSO_4_ is vasodilatator and hypotensive, with hypermagnesemia >4.5 mEq/L (2.5 mmol/L) in preterm infants (Ali et al. 2003). We previously demonstrated the expression of functional NMDA receptors by endothelial cells from neonatal brain microvessels (Henry et al. 2013; Porte et al. 2017). Thus, we investigated the effects of MgSO_4_ on calcium activity, vascular plasticity, and cell survival in neonatal endothelial cells. Major differences were found between neurons and endothelial cells. Whereas in neurons 1‐mmol/L and 6.5‐mmol/L concentrations of MgSO_4_ were similarly neuroprotective, in the vascular system in vivo and in vitro experiments revealed a marked and significant dose‐dependent effect of MgSO_4_ on brain angiogenesis, vessel suffering, calcium mobilization, and endothelial cell survival. Similarly, a dose effect was found at a hemodynamic level. Laser speckle experiments showed that the highest neuroprotective dose of MgSO_4_ induced a reduction of the brain blood flux indicative of hypoperfusion in the fetal brain, whereas the lowest neuroprotective dose of MgSO_4_ did not. Finally, at a molecular level, a dose effect of MgSO_4_ was also found regarding VEGF‐R2 expression and Hif‐1*α* nuclear translocation. Consistent with these data, Hif‐1*α* is a hypoxia sensor and one of the most well‐studied and predominant effects of HIF‐induced transcription is the induction of VEGF and its receptors under hypoxic conditions (Marti et al. 2000). In addition, it has been shown that VEGF‐R2 plays a key role in the sprouting and branching of vessels by regulating the expression of the Notch ligand DLL4 (de Bock et al. 2013). Altogether, these data indicate, for the first time, that a high dose of MgSO_4_ impairs brain angiogenesis in the mouse and the expression of pro‐angiogenic receptors from the VEGF family. They provide new highlights regarding the mechanistic pathways that would contribute to the side effects of high and cumulative doses of MgSO_4_ in the vascular system of the neonatal brain in humans.

The present study showed that MgSO_4_ induced neuroprotection against excitotoxicity in cultured brain slices, but also angiogenic/hemodynamic effects in vivo when increasing the injected dose. These results raised the question of the bioavailability into the brain of peripherally administered MgSO_4_. Experiments performed in animal models and/or in human showed that when blood–brain barrier is established, plasmatic hypermagnesemia only produced a marginal increase in magnesium levels in the cerebrospinal fluid suggesting a low bioavailability of MgSO_4_ to the mature brain (McKee et al. 2005). In contrast, even if studies investigating the bioavailability of MgSO_4_ in the fetal brain are scarce, a preclinical study from Hallak's group showed that MgSO_4_ injected subcutaneously in pregnant rats crosses the placenta within 2 h, enters the fetal brain, and concentrates into the forebrain (Hallak and Cotton 1993). These data suggest age‐dependent brain availability of MgSO_4_ when it is peripherally administered.

Our preclinical data showed that, in the fetal mouse brain, MgSO_4_ induced dose‐dependent effects on cortical vasculature, on Hif‐1*α* stabilization as well as on the expression of proteins involved in angiogenesis. In human, several protocols are used worldwide with MgSO_4_ for neuroprotection and prevention of cerebral palsy (Doyle et al. 2007; Rouse et al. 2008; Chollat et al. 2014) or treatment of preeclampsia (Ueda et al. 2016). In particular, studies performed in humans in the context of preeclampsia showed that loading doses of MgSO_4_ ranging from 6 to 14 g significantly affected hemodynamic parameters in the middle cerebral artery (MCA) of the fetus (Souza et al. 2010; Farshchian et al. 2012). In particular, Souza and co‐workers showed that 6‐g bolus of MgSO_4_ administered within 20 min to pregnant women significantly reduced Doppler indexes, such as the resistance index (−4%; 95% CI, 0.03; *P *<* *0.001), pulsatility index (−10%; 95% CI, 0.15; *P *<* *0.001), and systolic/diastolic ratio (−13%; 95% CI, 0.55; *P *<* *0.001) in the MCA of the fetus (Souza et al. 2010). These data support that MgSO_4_ is able to impact hemodynamic parameters of the human fetal brain. Based on these statements, we performed a Doppler analysis of the MCA of fetuses to evaluate the hemodynamic effects of a 4‐g loading dose of MgSO_4_ administered to women in the context of neuroprotection. In contrast to the 6‐g and 14‐g MgSO_4_ loading doses, we found no effect of MgSO_4_ on Doppler indexes quantified before and during administration of 4‐g MgSO_4_ supporting that hemodynamic effects of MgSO_4_ in the human fetal brain are dose‐dependent. However, retrospective analysis constitutes a limitation for the interpretation of clinical data. Consequently and in order to overcome this limitation, we also performed in vivo laser speckle acquisitions in fetal brains from pregnant mice exposed to different doses of MgSO_4_. Interestingly, results showed that increasing MgSO_4_ concentration was associated with an increase in brain hemodynamic alterations supporting clinical data as well as the retrospective analysis.

In addition to loading doses, a strong variability of MgSO_4_ regimens is described in the literature regarding maintenance doses and treatment durations. For example, in the PREMAG trial, women received a single 4‐g loading dose without maintenance (Marret et al. 2007). In the ACTOMgSO4 trial, women received a 4‐g loading dose followed by a maintenance infusion of 1 g/h for up to 24 h (Crowther et al.*,* 2003), whereas in the trial published by Rouse and co‐workers, women received a 6‐g bolus followed by a constant infusion of 2 g/h during 12 h (Rouse et al. 2008). Consequently, total dose of MgSO_4_ received by the fetus can markedly change from one trial to another one. Several studies reported possible adverse effects, including vasculopathies on fetuses from women treated for severe preeclampsia and whose received high doses of MgSO_4_ (Abbassi‐Ghanavati et al. 2012; De Jesus et al. 2015). Altogether, these data raise the question of the added value of long periods of MgSO_4_ maintenance doses for the fetus in particular in case of preeclampsia.

Even though, for reasons of metabolic specificities, it is important to be cautious when comparing doses used in animal models and in clinic (Perrin 2014). For an injection of 270 mg/kg of MgSO_4_ to pregnant rats, Hallak and co‐workers found a blood peak of magnesium at 1.52 mmol/L after 30 min (Hallak et al. 2000). Regarding the highest tested dose of MgSO_4_ (600 mg/kg), a time course experiment showed a serum peak at 2.5 mmol/L after 30 min (Wolf et al. 1991). In the present study, we compared, for the first time, the vascular effects of graded neuroprotective doses of MgSO_4_ ranging from 100 to 600 mg/kg. These doses were chosen to include those previously used in the literature (Wolf et al. 1991; Hallak et al. 2000). Our data clearly showed angiogenic effects for the high (600 mg/kg), but not for the low (100 mg/kg), dose of MgSO_4_. Interestingly, a retrospective cohort study showed that clinical outcomes were found in a subset of preterm human neonates with serum magnesium levels >2.3 mmol/L (Basu et al. 2012). Moreover, in this clinical study, mothers received a loading dose of 6‐g MgSO_4_ infused over 30 min, followed by a maintenance infusion of 2 g/h until the time of delivery. Despite evident precautions regarding species transposition, these values are consistent with those found in the blood of animals (2.5 mmol/L) treated with the high dose of MgSO_4_ (600 mg/kg; (Wolf et al. 1991).

In conclusion, using preclinical approaches, the present study provides the first mechanistic evidence that MgSO_4_ differently impacts the activity and survival of neuronal and endothelial cells in the fetal brain. In particular, double‐sword and dose‐dependent actions of MgSO_4_ on neuroprotection and angiogenesis were revealed. At a clinical level, these results indicated that, in contrast to higher loading doses, a 4‐g MgSO_4_ bolus does not induce adverse effects on brain hemodynamic parameters in the fetus. These data support the use of neuroprotection protocols with a 4‐g MgSO_4_ loading dose and a maintenance regimen limited to 1 or 2 g/h during 12 h to limit the cumulative dose.

## Author Contributions

Participated in research design: Gonzalez, Marret, Vivien. Conducted experiments: Chollat, Lecuyer, Lecointre, Rubio. Performed data analysis: Chollat, Gonzalez, Lecuyer, Marret. Wrote and contributed to the writing of the manuscript: All authors.

## Disclosure

None declared.

## Supporting information


**Data S1.** Supplementary Results.
**Figure S1.** Effects of graded concentrations of MgSO_4_ on indicators of apoptotic death under basal and excitotoxic conditions.
**Table S1.** Origin and characteristics of the antibodies used for immunohistochemical and Western blot studies.
**Table S2.** Sequences of the primers used for quantitative RT‐PCR experiments.
**Table S3.** Statistical analysis data.
