# Supplementary material for: Experimental and clinical evidence of differential effects of magnesium sulfate on neuroprotection and angiogenesis in the fetal brain
Source: Pharmacol Res Perspect. 2017 Jun 8;5(4):e00315. doi: 10.1002/prp2.315 (PMC5684858; doi:10.1002/prp2.315)
Supplement: Supplementary file 1 — Data S1. Supplementary Results. Figure S1. Effects of graded concentrations of MgSO4 on indicators of apoptotic death under basal and excitotoxic conditions. Table S1. Origin and characteristics of the antibodies used for immunohistochemical and Western blot studies. Table S2. Sequences of the primers used for quantitative RT‐PCR experiments. Table S3. Statistical analysis data. [file PRP2-5-e00315-s001.docx]

**Supplementary Material for Review**

***Supplementary Results***

***Graded doses of MgSO4 have no side effects on apoptotic cell death in brain slices cultured under basal and excitotoxic conditions***

Several pre-clinical studies revealed that blockade of the NMDA receptor is pro-apoptotic and deleterious for the developing brain (Aligny et al., 2014; Hansen et al., 2004). To determine whether MgSO_4_ alone would impact apoptotic cell death, we investigated the effects of a graded concentration of MgSO_4_ on caspase-3 activity, caspase-3 cleavage, and Bax expression in cultured brain slices from mouse neonates (Supplementary Fig. 1). Six-hour treatment of slices with MgSO_4_ in doses ranging from 1 to 8 mM had no effect on caspase-3 activity (Supplementary Fig. 1A). The same experiment performed with MK801 (20 µM) and glutamate (400 µM), used as positive and negative controls, resulted in a marked increase (p<0.0001) and a significant (p<0.0001) decrease of caspase-3 activity, respectively (Supplementary Fig. 1A). Immunohistochemistry experiments showed that cleaved caspase-3 labeling was preferentially localized in superficial cortical layers II to IV (Supplementary Fig. 1B-E). Although MgSO_4_ did not modify the pattern of cleaved caspase-3 immunolabeling (Supplementary Fig. 1C), MK801 and glutamate, respectively, increased and reduced caspase-3 immunoreactivity in developing cortical layers II to IV (Supplementary Fig. 1D, E). We also investigated the effects of co-incubation of low (1 mM) and high (6.5 mM) concentrations of MgSO_4_ in the presence of glutamate (400 µM; Supplementary Fig. 1F). Regardless of the dose tested, co-treatment of brain slices with MgSO_4_ and glutamate did not modify caspase-3 activity when compared to the control condition (Supplementary Fig. 1F). Western blot experiments indicated that, in contrast to the NMDA antagonist MK801, which significantly (p<0.05) increased caspase-3 cleavage, low and high concentrations of MgSO_4_ had no effects on the processing of caspase-3 (Supplementary Fig. 1G). Similar results were obtained concerning the expression levels of the pro-apoptotic protein Bax (Supplementary Fig. 1H). Immunohistochemistry confirmed that induction of Bax after MK801 (20 µM) treatment was localized in the same layers (II-IV) as observed for cleaved caspase-3 (Supplementary Fig. 1 I-L). Altogether, these data indicate that, in contrast to the NMDA antagonist MK801, low (1mM) and high (6.5 mM) doses of MgSO_4_ are not pro-apoptotic in the developing cortex of mouse neonates.

**Legend of Supplementary Fig. 1.** Effects of graded concentrations of MgSO_4_ on indicators of apoptotic death under basal and excitotoxic conditions. (A) Quantification of the caspase-3 activity after 6-hour incubation of cortex slices from P2 neonates in the absence (Ctrl) or presence of graded concentrations (1-8 mM) of MgSO_4_. The NMDA antagonist MK801 (20 µM) and glutamate (400 µM) were used as positive and negative controls, respectively. One-way ANOVA test showed a group effect (F=11.06; p<0.0001) and Dunnett’s multiple comparison test indicated ****p < 0.0001 *vs* control. (B-E) Visualization by immunohistochemistry of the cleaved caspase-3 labeling in brain slices from P2 neonates after 6-hour incubation in the absence (B) or presence of 1 mM MgSO_4_ (C), MK801 (20 µM; D) and glutamate (400 µM; E). Note that cleaved caspase-3 immunoreactivity is preferentially localized in superficial layers II to IV. (F) Quantification of caspase-3 activity under excitotoxic conditions. Cortex slices were incubated for 6 hours with glutamate (400 µM) in the absence or presence of low (1 mM) and high (6.5 mM) concentrations of MgSO_4_ or MK801 (20 µM). One-way ANOVA test showed a group effect (F=17.07; P<0.0001) and Tukey’s multiple comparison test indicated the following: **p<0.01 *vs* control; ####p<0.0001 *vs* glutamate. (G) Effects of low and high concentrations of MgSO_4_ on cleaved caspase-3 levels after 6-hour incubation of P2 cortical slices with low (1 mM) and high (6.5 mM) concentrations of MgSO_4_. MK801 (20 µM) was used as a positive control. *p<0.05 *vs* control using unpaired t test. (H) Quantification by Western blot of Bax expression after 6-hour incubation of P2 cortical slices with low (1 mM) and high (6.5 mM) concentrations of MgSO_4_. MK801 (20 µM) was used as a positive control. ***p<0.001 *vs* control using unpaired t test. (I-K) Visualization by immunohistochemistry of the pro-apoptotic protein Bax in the superficial layers of the neocortex from P2 neonates in control conditions (I) or after 6-hour incubation with MK801 (20 µM; J) and MgSO_4_ (1 mM; K). Note that the increase of Bax immunolabeling after MK801 treatment occurs on the superficial layers of the cortex. I–VI, cortical layers; CC, corpus callosum. Insert: Area detailed in (G). (L) Visualization at high magnification of Bax immunolabeling in superficial layers II to IV. Note the cytosolic localization of the fluorescent signal (arrow).

**Supplementary Table 1.** Origin and characteristics of the antibodies used for immunohistochemical and Western blot studies.

| **Antibodies** | **Trade reference** | **Supplier** | **Dilution** | **Solution of incubation** |
| --- | --- | --- | --- | --- |
| Hif 1-α | WB: NB100-479 | Novus Biologicals | 1/1000 | Milk (5% in TBST) |
|  | IHC: NB100-479 |  | 1/300 | BSA (1% in PBS) |
| PECAM/CD31 | WB: (M-20)  sc-1506 | Santa Cruz | 1/500 | BSA (5% in TBST) |
| VEGF-R1/Flt-1 | WB: (C-17) sc-316 | Santa Cruz | 1/1000 | Milk (5% in TBST) |
| VEGF-R2/Flk-1 | WB: (N-931) sc-505 | Santa Cruz | 1/1000 | Milk (5% in TBST) |
| Cleaved  caspase-3 | IHC: 9661 | Cell Signaling Technology | 1/200 | BSA (1% in PBS) |
| Caspase-3 | WB: 9665 | Cell Signaling Technology | 1/1000 | Milk (5% in TBST) |
| Bax | HIC: Sc-493 | Santa Cruz | 1/200 | BSA (1% in PBS) |
|  | WB: (N-20) Sc-493 |  | 1/500 | BSA (5% in TBST) |
| β-Actin | WB: A5441 | Sigma- Aldrich | 1/5000 | Milk (5% in TBST) |

**Supplementary Table 2.** Sequences of the primers used for quantitative RT-PCR experiments.

|  | **Gene-specific forward primer** | **Gene-specific reverse primer** |
| --- | --- | --- |
| **GAPDH** | 5’-TCATGGCCTTCCGTGTTCCTA-3’ | 5’-CCTGCTTCACCACCTTCTTGA-3 |
| **PECAM** | 5’-TCCAACAGAGCCAGCAGTATGA-3’ | 5’-TCCAATGACAACCACCGCAATG-3’ |
| **VEGF-R1** | 5’-GGGTCCTCGTTCCAGTCTTTC-3 | 5ʹ-CGGCTGCTTCCGATGTTT-3’ |
| **VEGF-R2** | 5’-GAGCCACATGGTCTCTCTGGTT-3ʹ | 5’-TGGTAGGAATCCATAGGCGAGAT-3’ |

**Supplementary Table 3.** Statistical analysis data

| **Experiments** | **Test** | **n** | **p value** | ***p<0.05**  ****p<0.01**  *****p<0.001**  ******p<0.0001** |
| --- | --- | --- | --- | --- |
| **LDH activity dose effect (Fig 1A)** | One-way ANOVA,  Dunnett's multiple comparison test | n=7  4 pooled hemi-cortices per sample | ANOVA: F=14.14,  p<0.0001  Dunnett's multiple comparisons test:  **Ct *vs* MgSO_4_(3.5mM)** p=0.0244  **Ct *vs* 6.5mM** p=0.0040  **Ct *vs* 8mM**  p=0.0005  **Ct *vs* MK801**  p<0.0001  **Ct *vs* Glut**  p<0.0001 | ******p<0.0001**  ***p<0.05**  ****p<0.01**  *****p<0.001**  ******p<0.0001**  ******p<0.0001** |
| **LDH activity combination (Fig 1F)** | One-way ANOVA,  Tukey's multiple comparison test | n=7  4 pooled hemi-cortices per sample | ANOVA: F=8.911,  p<0.0001  Tukey's multiple comparisons test:  **Ct *vs* MK801**  p=0.0422  **Ct *vs* Glut**  p=0.0186  **Glut *vs* Glut.Mk801**  p<0.0001  **Glut *vs* Glut.MgSO_4_ (1 mM)**  p=0.0003  **Glut *vs* Glut.MgSO_4_ (6.5 mM)**  p<0.0001 | ******p<0.0001**  ***p<0.05**  ****p<0.01**  **^####^p<0.0001**  **^###^p<0.001**  **^####^p<0.0001** |
| **Cell swelling**  **(Fig 1I)** | Two-way ANOVA,  Tukey's multiple comparisons test | 60 cells | Two-way ANOVA:  F=17.19, p<0.0001  Tukey's multiple comparisons test:  **aCSF *vs* Glut**  t=2h30, p<0.0001  t=3h00, p<0.0001  t=3h30, p<0.0001  t=4h00, p<0.0001  t=4h30, p<0.0001  t=5h00, p<0.0001  t=5h30, p<0.0001  t=6h00, p<0.0001  **aCSF *vs* MgSO_4_(1 mM)**  t=2h30, p=0.0372  t=3h00, p=0.0011  t=3h30, p<0.0001  t=4h00, p<0.0001  t=4h30, p<0.0001  t=5h00, p<0.0001  t=5h30, p<0.0001  t=6h00, p<0.0001  **aCSF *vs* Glut.MgSO_4_**  **(1 mM)**  t=2h30, p=0.0043  t=3h00, p=0.0011  t=3h30, p<0.0001  t=4h00, p<0.0001  t=4h30, p=0.0012  t=5h00, p=0.0005  t=5h30, p=0.0012  t=6h00, p=0.0010  **Glut *vs* Glut.MgSO_4_**  **(1 mM)**  t=2h00, p=0.0028  t=2h30, p=0.0043  t=3h00, p=0.0011  t=3h30, p<0.0001  t=4h00, p<0.0001  t=4h30, p<0.0001  t=5h00, p<0.0001  t=5h30, p<0.0001  t=6h00, p<00001 | ******p<0.0001**  ******p<0.0001**  ******p<0.0001**  ******p<0.0001**  ******p<0.0001**  ******p<0.0001**  ******p<0.0001**  ******p<0.0001**  ***p<0.05**  ****p<0.01**  ******p<0.0001**  ******p<0.0001**  ******p<0.0001**  ******p<0.0001**  ******p<0.0001**  ******p<0.0001**  ****p<0.01**  ******p<0.0001**  ******p<0.0001**  ******p<0.0001**  ****p<0.01**  *****p<0.001**  ****p<0.01**  ****p<0.01**  **^##^p<0.01**  **^####^p<0.0001**  **^####^p<0.0001**  **^####^p<0.0001**  **^####^p<0.0001**  **^####^p<0.0001**  **^####^p<0.0001**  **^####^p<0.0001**  **^####^p<0.0001** |
| **Survival curve**  **(Fig 1J)** | Log-rank (Mantel-Cox) test,  Chi square | 60 cells at the beginning from 3 independent slices | **aCSF *vs* Glut.MgSO_4_**  **χ^2^**= 81.95, p<0.0001  **aCSF *vs* MgSO_4_(1mM)**  **χ^2^**= 33.41, p<0.0001  **Glut *vs* Glut.MgSO_4_(1mM)**  **χ^2^**= 220.2, p<0.0001 | ******p<0.0001**  ******p<0.0001**  ******p<0.0001** |
| **Calcimetry neurons, AUC (Fig 2F and 2G)** | One-way ANOVA,  Dunnett's multiple comparison test | 20 cells per group from 3 independent slices | **Superficial layers**  **(Fig 5F)**  ANOVA: F=3.251,  p=0.0088  Dunnett's multiple comparison test:  **aCSF *vs* Glut**  p=0.0061  **Glut *vs* GlutMgSO_4_ (1 mM**)  p=0.0043  **Glut *vs* GlutMgSO_4_ (6.5 mM**)  p=0.0478  **Deep layers**  **(Fig 5G)**  ANOVA: F=47.24,  p<0.0001  Dunnett's multiple comparison test:  **aCSF *vs* Glut**  p<0.0001  **Glut *vs* Glut.MgSO_4_ (1 mM**)  p<0.0001  **Glut *vs* Glut.MgSO_4_ (6.5 mM**)  p<0.0001 | ****p<0.01**  ****p<0.01**  **^##^p<0.01**  **^#^p<0.05**  ******p<0.0001**  **^####^p<0.0001**  **^####^p<0.0001**  **^####^p<0.0001** |
| **Calcimetry endothelial cells AUC**  **(Fig 3G)** | One-way ANOVA,  Dunnett's multiple comparison test | 5 cells per group from 3 independent slices | ANOVA: F=5.540,  p=0.0016  Dunnett's multiple comparison test  **aCSF *vs* Glut**  p= 0.0027  **aCSF *vs* GlutMgSO_4_ (1 mM**)  p= 0.0321  **Glut *vs* Glut.MgSO_4_ (6.5 mM)**  p=0.0446 | ****p<0.01**  ****p<0.01**  ***p<0.05**  **^#^p<0.05** |
| **Micro-vessels remodeling (Fig 4C)** | Two-way ANOVA, Tukey's multiple comparisons test | 15 vessels per group from 3 independent slices | Two-way ANOVA: F=(24, 78) = 1.343,  p=0.1662  Tukey's multiple comparisons test:  ***Vs* MgSO_4_(6.5 mM)**  t=2h30, p= 0.0268  t=3h00, p=0.0033  t=3h30, p < 0.0002  t=4h00, p < 0.0003  t=4h30, p < 0.0003  t=5h00, p < 0.0005  t=5h30, p < 0.0007  t=6h00, p < 0.0007 | **ns**  ***p<0.05**  ****p<0.01**  *****p<0.001**  *****p<0.001**  *****p<0.001**  *****p<0.001**  *****p<0.001**  *****p<0.001** |
| **Endothelial mortality**  **(Fig 4E)** | One-way ANOVA,  Tukey's multiple comparisons test (*compared to control) | 10 endothelial cells from 5 independent slices | ANOVA: F=3.242,  p=0.0547  **Ctrl *vs* MgSO_4_(6.5mM)**  p<0.0436 | **ns**  ***p<0.05** |
| **Cortical vessels orientation (Fig 5C)** | Chi-square test | 2 pictures/slices; 3 slices/P2; 3P2/pregnant mice; more than 5 pregnant mice/condition | **NaCl *vs* MgSO_4_ (100 mg/kg)** chi-square: df=1090.1, p=0.2965  **NaCl *vs* MgSO_4_ (200 mg/kg)** chi-square: df=2913.1, p= 0.0878  **NaCl *vs* MgSO_4_ (600 mg/kg)** chi-square: df= 5548.1, p= 0.0185  **NaCl *vs* MgSO_4_ (800 mg/kg)** chi-square: df=4421.1, p= 0.0355 | **ns**  **ns**  ***p<0.05**  ***p<0.05** |
| **Cortical vessels diameter**  **(Fig 5D)** | One-way ANOVA,  Tukey's multiple comparisons test | 2 pictures/slice; 3 slices/P2; 3 P2/pregnant mice; more than 5 pregnant mice/condition | ANOVA: F=13.20 p<0.0001  Tukey's multiple comparisons test:  **NaCl *vs* MgSO_4_ (600 mg/kg)**  p<0.0001  **MgSO_4_ (100 mg/kg) *vs* (600 mg/kg)**  p= 0.0009 | ******p<0.0001**  ******p<0.0001**  **###p<0.001** |
| **qRT-PCR**  **VEGF-R1**  **(Fig 5E)** | One-way ANOVA,  Tukey's multiple comparisons test | 7 embryos per group | ANOVA: F=24.95  p<0.0001  Tukey's multiple comparisons test:  **NaCl *vs* MgSO_4_ (100 mg/kg)**  p=0.0003  **NaCl *vs* MgSO_4_ (600 mg/kg)**  p<0.0001 | ***p<0.05**  *****p<0.001**  ******p<0.0001** |
| **WB VEGF-R1**  **(Fig 5F)** | One-way ANOVA,  Tukey's multiple comparisons test (*compared to NaCl exposition) | 5 embryos per group | ANOVA: F=3.707,  p=0.0329  Tukey's multiple comparisons test:  **NaCl *vs* MgSO_4_ (600 mg/kg)**  p=0.0459 | ***p<0.05**  ***p<0.05** |
| **qRT-PCR VEGF-R2**  **(Fig 5G)** | One-way ANOVA,  Tukey's multiple comparisons test | 7 embryos per group | ANOVA: F=5.072,  p=0.0165  Tukey's multiple comparisons test:  **NaCl *vs* MgSO_4_ (600 mg/kg)**  p=0.0124 | ***p<0.05**  ***p<0.05** |
| **WB VEGF-R2**  **(Fig 5H)** | One-way ANOVA,  Tukey's multiple comparisons test | 5 embryos per group | ANOVA: F=3.539,  p=0.0380  Tukey's multiple comparisons test:  **NaCl *vs* MgSO_4_ (600 mg/kg)**  p=0.0476 | ***p<0.05**  ***p<0.05** |
| **qRT-PCR**  **PECAM**  **(Fig 5I)** | One-way ANOVA,  Tukey's multiple comparisons test | 7 embryos per group | ANOVA: F=4.348, p=0.0288  Tukey's multiple comparisons test:  **NaCl *vs* MgSO_4_ (600 mg/kg)**  p=0.0343 | ***p<0.05**  ***p<0.05** |
| **WB PECAM**  **(Fig 5J)** | One-way ANOVA,  Tukey's multiple comparisons test | 5 embryos per group | ANOVA: F=0.3927,  p=0.6795  Tukey's multiple comparisons test | **ns**  **ns** |
| **Cerebral blood flow**  **(Fig 6G)** | One-way ANOVA,  Tukey's multiple comparisons test (*compared to control and ^#^compared to MgSO_4_ (100mg/kg) | 25 embryos per group | ANOVA: F 8.469,  p= 0.0005  Tukey's multiple comparisons test:  **NaCl *vs* MgSO_4_ (600 mg/kg)**  p= 0.0009  **MgSO_4_ (100 mg/kg) *vs* MgSO_4_ (600 mg/kg)**  p= 0.0041 | *****p<0.001**  *****p<0.001**  **^##^p<0.01** |
| **WB Hi1-α**  **(Fig 6K)** | One-way ANOVA,  Tukey's multiple comparisons test | 3 embryos per group | ANOVA: F=7.908  p=0.0208  Tukey's multiple comparisons test:  **Ctrl *vs* MgSO_4_ (600 mg/kg)**  p=0,0436  **MgSO_4_ (100 mg/kg) *vs* MgSO_4_ (600 mg/kg)**  p=0.0247 | ***p<0.05**  ***p<0.05**  ***p<0.05** |
| **Human Doppler**  **(Fig 7)** | Paired t test | 27 cases | PSV: p=0.5530  PI: p=0.1922  RI: p=0.6858 | **ns**  **ns**  **ns** |
| **Caspase-3 activity dose effect (Suppl. Fig 1A)** | One-way ANOVA,  Dunnett's multiple comparison test | n=7  4 pooled hemi-cortices per sample | ANOVA: F=11.06.  p<0.0001  Dunnett's multiple comparisons test:  **Ct *vs* MK801**  p<0.0001  **Ct *vs* Glut**  p<0.0001 | ******p<0.0001**  ******p<0.0001**  ******p<0.0001** |
| **Caspase-3 activity combination (Suppl. Fig 1F)** | One-way ANOVA,  Tukey's multiple comparison test | n=7  4 pooled hemi-cortices per sample | ANOVA: F=17.07,  p<0.0001  Tukey's multiple comparisons test:  **Ct *vs* MK801**  p=0.0025  **Ct *vs* Glut**  p=0.0067  **Glut *vs* Glut.Mk801**  p<0.0001 | ******p<0.0001**  ****p<0.01**  ****p<0.01**  **^####^p<0.0001** |
| **WB cleaved caspase3**  **(Suppl. Fig 1G)** | Unpaired t test | n=5 | Unpaired t test:  p=0.0497  **Ct *vs* Mk801**  p=0.0497 | ***p<0.05**  ***p<0.05** |
| **WB Bax**  **(Suppl. Fig 1H)** | Unpaired t test | n=5 |  | *****p<0.001**  *****p<0.001** |
