# Supplementary figures and images for: Experimental and clinical evidence of differential effects of magnesium sulfate on neuroprotection and angiogenesis in the fetal brain
Source: Pharmacol Res Perspect. 2017 Jun 8;5(4):e00315. doi: 10.1002/prp2.315 (PMC5684858; doi:10.1002/prp2.315)

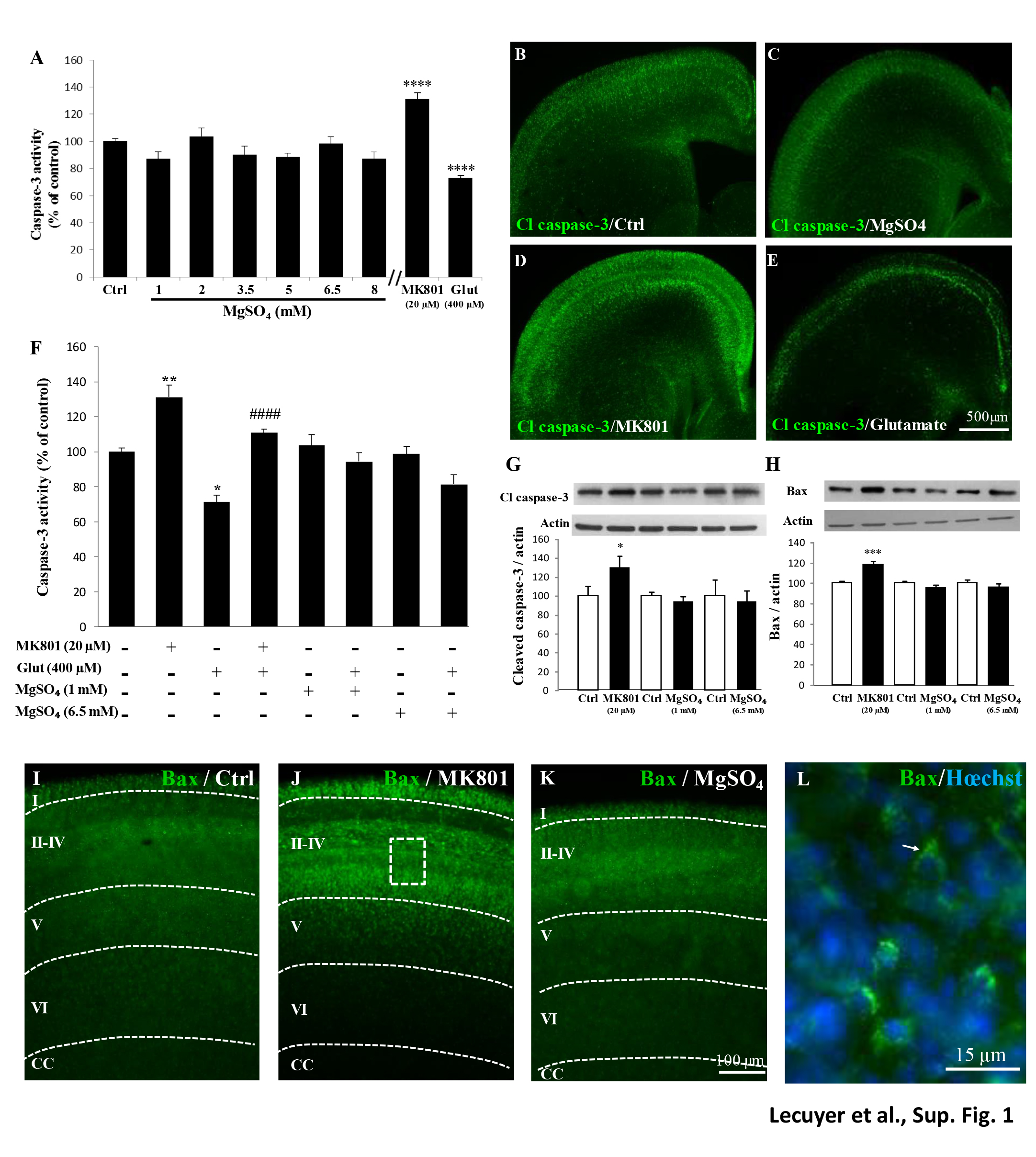

Supplement: Supplementary file 2 [file PRP2-5-e00315-s002.jpg]
